# Ubiquitylation is required for the incorporation of the Notch receptor into intraluminal vesicles to prevent prolonged and ligand-independent activation of the pathway

**DOI:** 10.1186/s12915-022-01245-y

**Published:** 2022-03-10

**Authors:** Björn Schnute, Hideyuki Shimizu, Marvin Lyga, Martin Baron, Thomas Klein

**Affiliations:** 1 Institute of Genetics, Heinrich-Heine-University Duesseldorf, Universitaetsstr. 1, 40225 Duesseldorf, Germany; 2 School of Biological Sciences, University of Manchester, Michael Smith Building, Oxford Road, Manchester, M13 9PT UK

**Keywords:** Notch signalling, Endocytosis, Ubiquitylation, E3-ligase, Su(dx), Dx, Itch

## Abstract

**Background:**

Ubiquitylation of the ligands and the receptor plays an important part in the regulation of the activity of the evolutionary conserved Notch signalling pathway. However, its function for activation of Notch is not completely understood, despite the identification of several E3 ligases devoted to the receptor.

**Results:**

Here we analysed a variant of the Notch receptor where all lysines in its intracellular domain are replaced by arginines. Our analysis of this variant revealed that ubiquitylation of Notch is not essential for its endocytosis. We identified two functions for ubiquitylation of lysines in the Notch receptor. First, it is required for the degradation of free Notch intracellular domain (NICD) in the nucleus, which prevents a prolonged activation of the pathway. More importantly, it is also required for the incorporation of Notch into intraluminal vesicles of maturing endosomes to prevent ligand-independent activation of the pathway from late endosomal compartments.

**Conclusions:**

The findings clarify the role of lysine-dependent ubiquitylation of the Notch receptor and indicate that Notch is endocytosed by several independent operating mechanisms.

**Supplementary Information:**

The online version contains supplementary material available at 10.1186/s12915-022-01245-y.

## Background

Notch (N) signalling is important for a diverse number of processes taking place in development, homeostasis and disease [1]. It mediates short-range signalling that requires direct contact between communicating cells, since the ligands and the N receptor are transmembrane proteins. The core pathway comprises the ligands, the receptor and a member of the CSL transcription factor family [2]. In *Drosophila*, it is initiated by binding of one of the two ligands in the genome, encoded by *Delta (Dl)* and *Serrate (Ser)*, to the N receptor. It elicits two sequential cleavages mediated by Kuzbanian (Kuz), the *Drosophila* homologue of the metalloprotease ADAM10, and γ-secretase, respectively, to release the intracellular domain of N (NICD) into the cytosol. The first, Kuz-mediated, cleavage causes the shedding of the extracellular domain (ECD) and creates an intermediate that is termed NEXT (Notch Extracellular Truncated). Ecto-domain shedding of Notch enables the intermembrane S3-cleavage by γ-secretase to release NICD. After entering the nucleus, NICD acts as a co-factor of CSL to activate the expression of target genes.

The endosomal pathway is required in several ways for the regulation of the activity of the N pathway. During activation of N, it creates a pulling force that induces a conformational change, which allows the access of Kuz to its cleavage site [3–5]. It was thought that endocytosis is initiated by ubiquitylation (ubi) and usually occurs on lysines (Ks). This notion was transferred from other examples of transmembrane proteins, such as the receptor tyrosine kinases [6]. Support for a role of ubi in the initiation of endocytosis of N came from the identification of three E3 ligases that can ubiquitylate the ICD of Notch and also initiate endocytosis, termed Deltex (Dx), Nedd4, and Suppressor of deltex (Su(dx)) [7]. Moreover, Nedd4 can ubiquitylate the ICD of N [8]. However, it is now clear that endocytosis of EGF-receptor can also be initiated in an ubi-independent manner [9–11]. This raises the question whether ubi is essential for the endocytosis of N.

Previous work showed that N is continuously internalised by Clathrin-mediated endocytosis independently of its activation, to assure the presence of functional receptors on the cell surface [12, 13]. On its way to the lysosome, N is concentrated and then incorporated into vesicles abutting from the limiting membrane (LM) of the maturing endosome (ME) into its lumen [10]. The incorporation into these intraluminal vesicles (ILVs) assures the complete presence of N in the lumen of the ME, which is a prerequisite for the complete degradation upon fusion with the lysosome. ILVs are formed by the ESCRT machinery, which consists of five in sequence acting complexes, ESCRT-0, I, II, III and the Vps4 AAA ATPase complex [14]. The events are initiated by ESCRT-0 which binds to the endosome-specific phosphatidylinositol 3-phosphate (PI(3)P). PI(3)P is indirectly generated by the activity of the early endosomal organiser Rab5 via recruitment of the PI 3-kinase Vps34. ESCRT-0 concentrates the cargo destined to be incorporated at the spot of ILV formation. To be recognised by the early acting ESCRT complexes, the cargo must be poly-ubiquitylated [10, 15]. Thus, ubi of the ICD is the prerequisite for incorporation of cargo, such as Notch, into ILVs.

Based on the analysis of several mutant situations, e.g. ESCRT or *lethal (2) giant discs* (*lgd*) mutants, it has been suggested that the failure of incorporation of N into ILVs causes the observed ectopic and ligand-independent activation of the N pathway [10, 16]. As a consequence of the failure of incorporation into ILVs, N remains in the LM of the ME and only the ECD of N reaches into the lumen upon fusion with the lysosome. Consequently, only the ECD can be degraded after fusion of the ME with the lysosome by activated acidic hydrolases. The degradation of the ECD, hereafter referred to as alternative ecto-domain shedding, creates an intermediate that resembles NEXT and is a substrate of γ-secretase. The released NICD travels to the nucleus to activate the target genes. Although this model of alternative ecto-domain shedding is plausible, it has never been tested whether a N that remains in the LM of the ME is really activated.

Recent work indicates that Dl can signal weakly in the absence of ubi of its intracellular domain [17]. Moreover, Su(dx) appears to induce endocytosis of N, even if they lack the ubi-mediating HECT domain [18–20]. These results suggest that ubi is not an absolute requirement for endocytosis of Dl or N and raises the question about the function of ubi during regulation of the activity of the pathway. Recent work has also discovered a second function for Dx and Su(dx), which is the regulation of the amount of N that is incorporated into ILVs [19–21]. This is achieved by different ubi modes. Su(dx) is postulated to perform poly-ubi, a tag that can be recognised by the initiating ESCRT-0 [22]. The result of Dx activity is mono-ubi, which is not recognised by ESCRT-0 [15]. The antagonism by the two E3 ligases determines (1) different endocytic routes of N and (2) the amount of N incorporated into ILVs in a temperature-dependent manner [19]. By regulation of the amount of Notch remaining at the limiting membrane of the ME, it also determines the amount of Notch which is activated in a ligand-independent manner. However, this model is based on over-expression of the elements involved and requires further confirmation.

Ubi of transmembrane proteins by E3 ligases occurs on lysins (Ks) of their ICDs. To further investigate the meaning of ubi for the activity of N, we analysed a variant of N where all lysins (Ks) in its ICD are replaced by the structurally similar arginines (Rs). Our analysis identified two functions for the Ks and ubi of N in its ICD: It is required for the degradation of the released NICD in the nucleus, which prevents a pro-longed activation of the pathway. In addition, it is required for the incorporation of N into ILVs of MEs to prevent ligand-independent activation of the pathway from late endosomal compartments. However, the ubi of its ICD is not essential for endocytosis of N. The findings clarify the role of K-dependent ubi of the Notch receptor and also indicate that Notch is endocytosed by several independently operating mechanisms.

## Results

**Fig. 1:**
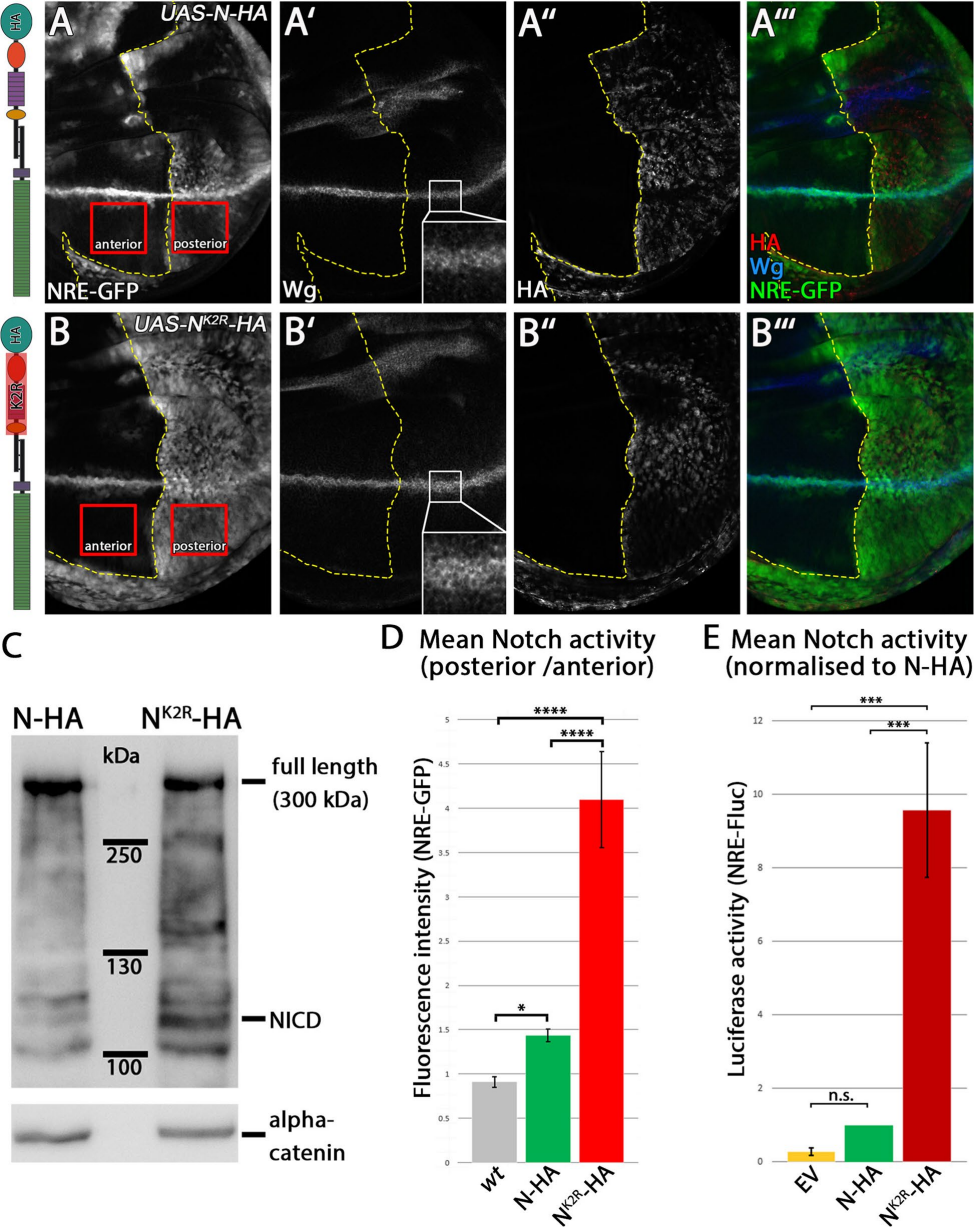
N^K2R^-HA induces ectopic activation of the N signalling pathway. **A–B”’** N-HA and N^K2R^-HA were expressed under control of *hh-GAL4, tub-GAL80ts* for 14.5 h in the posterior compartment of late third instar larvae wing imaginal discs. Activity of the N signalling pathway was indicated by the N target gene Wg and the activity reporter construct NRE-GFP. The expression of *hh-Gal4* is restricted to the right side of the stippled yellow line. The left side is the wild-type control. In contrast to N-HA, N^K2R^-HA induced a strong activation of NRE-GFP and a slight ectopic expression of Wg (**A**, **A’**, **B**, **B’**). **C** N-HA and NK2R-HA western blot analysis of whole larvae lysate. Transgenic N constructs were identified by HA antibody staining. In contrast to N-HA, N^K2R^-HA showed a strong accumulation of NICD. **D** Quantification of N activation in wing imaginal discs by fluorescence intensity measurement, shown in Fig. 1A, B. NRE-GFP intensity of the posterior compartment was normalised to the anterior compartment (wt: *n* = 9 / N-HA: *n* = 7 / N^K2R^-HA: *n* = 11, significance test: one-way ANOVA *p* ≥ 0.05 (n.s.) / *p* ≤ 0.05 (*) / *p* ≤ 0.01 (**) / *p* ≤ 0.001 (***) / *p* ≤ 0.0001 (****)). N^K2R^-HA induced a significantly stronger activation of the N-pathway than N-HA. **E** Quantification of N activation in an S2 cell luciferase-assay. Activation of the N signalling pathway was indicated by NRE-Fluc. NRE-Fluc fluorescence intensity was normalised to N-HA activity (*n* = 3 luciferase-assays, significance test: one-way ANOVA *p* ≥ 0.05 (n.s.) / *p* ≤ 0.05 (*) / *p* ≤ 0.01 (**) / *p* ≤ 0.001 (***) / *p* ≤ 0.0001 (****)). Basal N activation induced by N^K2R^-HA was significantly increased

To investigate the impact of ubi of the ICD of N on its signalling activity and trafficking, we replaced all 27 intracellular Ks by the structurally similar R (N^K2R^-HA). As control, we used a full-length N receptor (N-HA) (Fig. 1A, B). Both receptor variants are C-terminally tagged with HA and inserted into the same genomic landing site to guarantee comparable expression levels. Western blot analysis of the lysate harvested from late third instar larvae, where the variants were expressed for 24 h, showed a dominant band at approximately 300 kDa for both receptor variants, representing the full-length receptor of similar intensity (Fig. 1C). The band at 110 kDa, which represents NICD, is stronger for N^K2R^-HA than for N-HA, suggesting an increase in S3 cleavage of N^K2R^-HA compared to N-HA, or a higher stability of the resulting NICD^K2R^-HA, or both.

### The loss of the Ks in the ICD of N causes an increase of its activity

First, we tested the ability of N^K2R^-HA and N-HA to activate the N pathway in vivo. To do so, we monitored the expression of target genes in the wing imaginal disc upon expression of the variants with the Gal4 system. In contrast to N-HA, continuous expression of N^K2R^-HA with several Gal4 drivers resulted in lethality of the flies before the third larval instar stage. This was previously observed by us for the activated forms of N (NICD) and, together with the stronger NICD band in the Western blot analysis, suggested that N^K2R^-HA is an over-active variant of Notch. To avoid early lethality, we switched to the TARGET system, which allows temporal control of expression of the transgenes by temperature shifts [23]. Using TARGET, we expressed N-HA and N^K2R^-HA in a pulse of 14.5 h in the posterior compartment of third instar larvae wing imaginal discs (*hh-GAL4 tub-Gal80* ^*ts*^). We found that this temporally restricted expression of N^K2R^-HA allowed survival of the animals and induced a much stronger ectopic expression of the N activity reporter NRE-GFP than N-HA ([24], Fig. 1A–B”’). This observation confirmed that the activity of N^K2R^-HA is increased relative to N-HA. For better comparison of the activity of the two N-variants, we quantified the GFP signal of NRE-GFP in the manipulated posterior *hh-Gal4* expressing compartment and normalised it to the signal of the anterior control compartment (Fig. 1D, see M&M). This analysis confirmed that N^K2R^-HA is much more active than N-HA. Thus, loss of the Ks in its ICD increases the activity of N, instead of decreasing it.

### N^K2R^-HA can be activated in a ligand-dependent and ligand-independent manner

To explore the cause of the stronger activity of N^K2R^-HA, we tested whether the activation of our N variants depends on the presence of Dl and Ser, or is indirectly induced by activation of endogenous N. To do so, we first analysed their signalling capacity with an established luciferase assay that measures N activity in S2 cells [25]. S2 cells do not express endogenous N and Dl and only weakly Ser [26, 27]. The assay showed that, in contrast to N-HA, N^K2R^-HA expression resulted in a strong activation of NRE-Fluc, suggesting that it is activated ligand-independently (Fig. 1E).

**Fig. 2:**
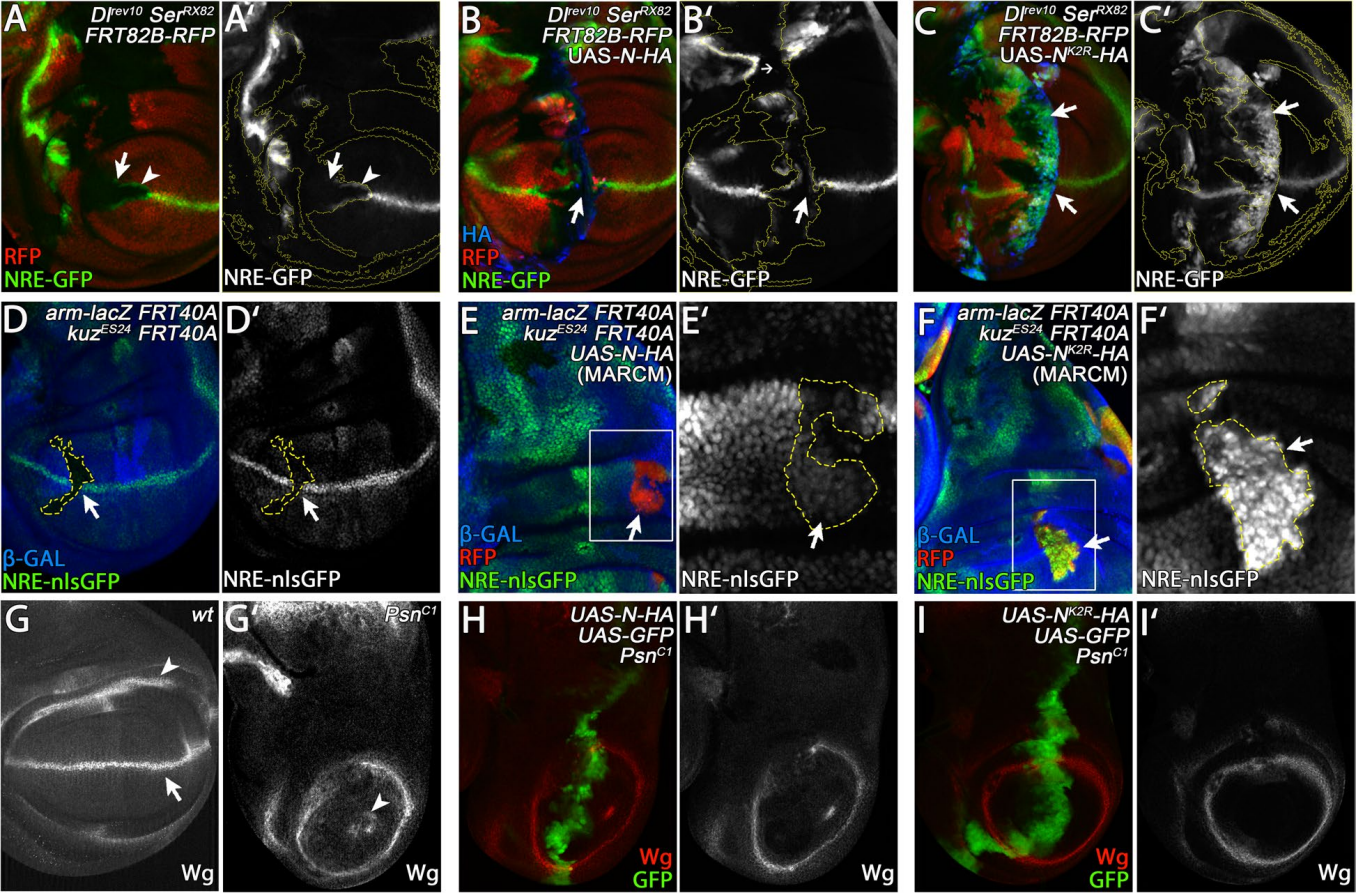
N^K2R^-HA induced N signalling is mediated ligand and Kuzbanian independent. **A–C’** Expression of N-HA and N^K2R^-HA in *Dl* ^*rev10*^ *Ser* ^*RX82*^ double mutant cell clones in late third instar wing imaginal discs. Double mutant cell clones are indicated by the absence of RFP. N-HA and N^K2R^-HA were expressed under control of *ptc-GAL4* at the A/P boundary. N signalling is detected by the expression of NRE-GFP. The clone boundaries in (**A’**, **B’**, **C’**) are outlined by the stippled yellow lines. **A**, **A’** Endogenous N signalling along the D/V boundary of the wing is abolished in double mutant cell clones (arrow). Due to relief of *cis*-inhibition, N signalling is induced in the *Dl Ser* double mutant cells at the clone boundary (arrowhead). **B**, **B’** Expression of N-HA is not sufficient to rescue the loss of N signalling in double mutant cell clones at the D/V boundary (arrow). **C**, **C’** In contrast to N-HA, N^K2R^-HA induces strong ectopic N signalling in *Ser Dl* double mutant cell clones, indicating that it acts in a ligand-independent manner (arrows). **D–F’** MARCM clones expressing N-HA or N^K2R^-HA in *kuz* ^*ES24*^ mutant cells. The *kuz* ^*ES24*^ clones are indicated by the absence of arm-lacZ (see also M&M) and the clone boundaries in **D’**, **E’**, **F’** are outlined by the stippled yellow lines. Expression of the constructs is indicated by RFP. Area magnified in **E’** and **F’** are highlighted by the white boxes in **E**, **F**. **D**, **D’** N signalling at the D/V boundary is abolished in *kuz* ^*ES24*^ mutant cell clones (arrow). **E**, **E’** N-HA was unable to induce ectopic N signalling in mutant cell clones (arrow), indicating that N-HA activation depends on the function of *kuz*. **F**, **F’** In contrast, expression of N^K2R^-HA induced strong expression of NRE-GFP in in the absence of *kuz* function. **G–H’** Expression of N-HA and N^K2R^-HA in *Psn* ^*C1*^ mutant wing imaginal discs. The variants were expressed under control of *ptc-GAL4*. **G**, **G’** *Psn* ^*C1*^ mutant wing imaginal discs display a loss of expression of the target Wg along the D/V boundary (arrow in **G**). Moreover, the inner ring-like domain of Wg expression (arrowhead in **G**) is reduced to a spot or is even absent (arrowhead in **G’**). Ectopic expression of N-HA (**H**, **H’**) or N^K2R^-HA (**I**, **I’**) did not induce Wg expression at the D/V boundary in the absence of *Psn* function, indicating the requirement of S3 cleavage for their activation of the Notch pathway

To investigate whether N^K2R^-HA is activated in a ligand-independent manner in vivo, we monitored the activity of N^K2R^-HA in the absence of *Dl* and *Ser* function in vivo in wing imaginal disc using clonal analysis. As expected, *Dl* ^*revF10*^, *Ser*^*RX22*^ double mutant cell clones caused a loss of expression of the Notch activity reporter NRE-GFP along the D/V boundary of the wing anlage (Fig. 2A, A’, arrow). The expression of N-HA in the double mutant clones failed to induce expression of NRE-GFP, indicating that its activation depends on the presence of the ligands (Fig. 2B, B’, arrow). In contrast, strong ectopic activation of NRE-GFP was induced by expression of N^K2R^-HA in *Dl*, *Ser* mutant clones (Fig. 2C, C’, arrows). These results confirm that, in contrast to N-HA, N^K2R^-HA is activated in a ligand-independent manner. This ligand-independent activity contributes to its higher activity.

In order to test whether N^K2R^-HA can be also activated by its ligands, we turned to S2 cells which do not express Notch and Dl (Fig. S1). In the assay, the N-variants were expressed alone, or together with Dl. The activity of the Notch pathway was revealed by a luciferase assay, using the Notch-sensitive NRE-luciferase construct. The assay revealed that the activity of N-HA and also N^K2R^-HA can be enhanced by the ligand. Hence, N^K2R^-HA is activated in a ligand-dependent and also ligand-independent manner. Note, that, in contrast to N-HA, the activity of N^K2R^-HA is only moderately increased by the addition of Dl, indicating that the ligand-independent activation contributes the most to the activity of N^K2R^-HA.

### The ligand-independent activation of N^K2R^-HA requires the S3-, but not the S2-cleavage

Next, we investigated the requirements for the observed ligand-independent activation of N^K2R^-HA. We first analysed if its activation depends on Kuz/ADAM10-mediated S2-cleavage. We therefore monitored the activity of N-HA and N^K2R^-HA in *kuz* ^*ES24*^ mutant MARCM cell clones. Loss of *kuz* function results in cell-autonomous loss of N signalling [28–31] (Fig. 2D, D’, arrow). When N-HA is expressed in *kuz*-mutant cell clones, N activation was abolished, indicating that the activity of N-HA depends on Kuz-mediated S2-cleavage (Fig. 2E, E’, arrow). In contrast, expression of N^K2R^-HA induced strong ectopic N signalling in *kuz* mutant cells (Fig. 2F, F’, arrow). Thus, N^K2R^-HA appears to be activated in a *kuz*-independent manner and therefore does not require S2 cleavage.

Next, we tested whether N^K2R^-HA induced N-signalling depends on γ-secretase-mediated S3-cleavage. For this purpose, we expressed N-HA or N^K2R^-HA in *Presenilin* (*Psn*) mutant wing imaginal discs. Psn is the catalytic subunit of the γ-secretase complex [32, 33]. Loss of *Psn* function causes the complete loss of N activity, here indicated by the loss of Wg expression along the D/V-boundary (Fig. 2G, G’, arrow in G). In contrast to wild-type discs, expression of N-HA and also N^K2R^-HA failed to induce Wg expression in *Psn* mutant discs, indicating that the activation of the N-pathway by N-HA and N^K2R^-HA requires the activity of the γ-secretase-mediated S3-cleavage (Fig. 2H–I’).

Previous work indicates that the γ-secretase can non-specifically cleave N-variants with an ECD fewer than 100 amino acids [34]. Thus, N^K2R^-HA probably undergoes alternative (Kuz-independent) ecto-domain shedding to become a substrate. Altogether, the results are compatible with the notion that N^K2R^-HA activates the Notch pathway in a ligand-independent manner by alternative ectodomain shedding and subsequent γ-secretase-mediated S3-cleavage. The requirements for activation resemble that for the ligand-independent activation of the pathway in *lgd* mutant cells [16, 35–37].

### Degradation of the cleaved ICD of N^K2R^-HA in the nucleus is delayed

**Fig. 3:**
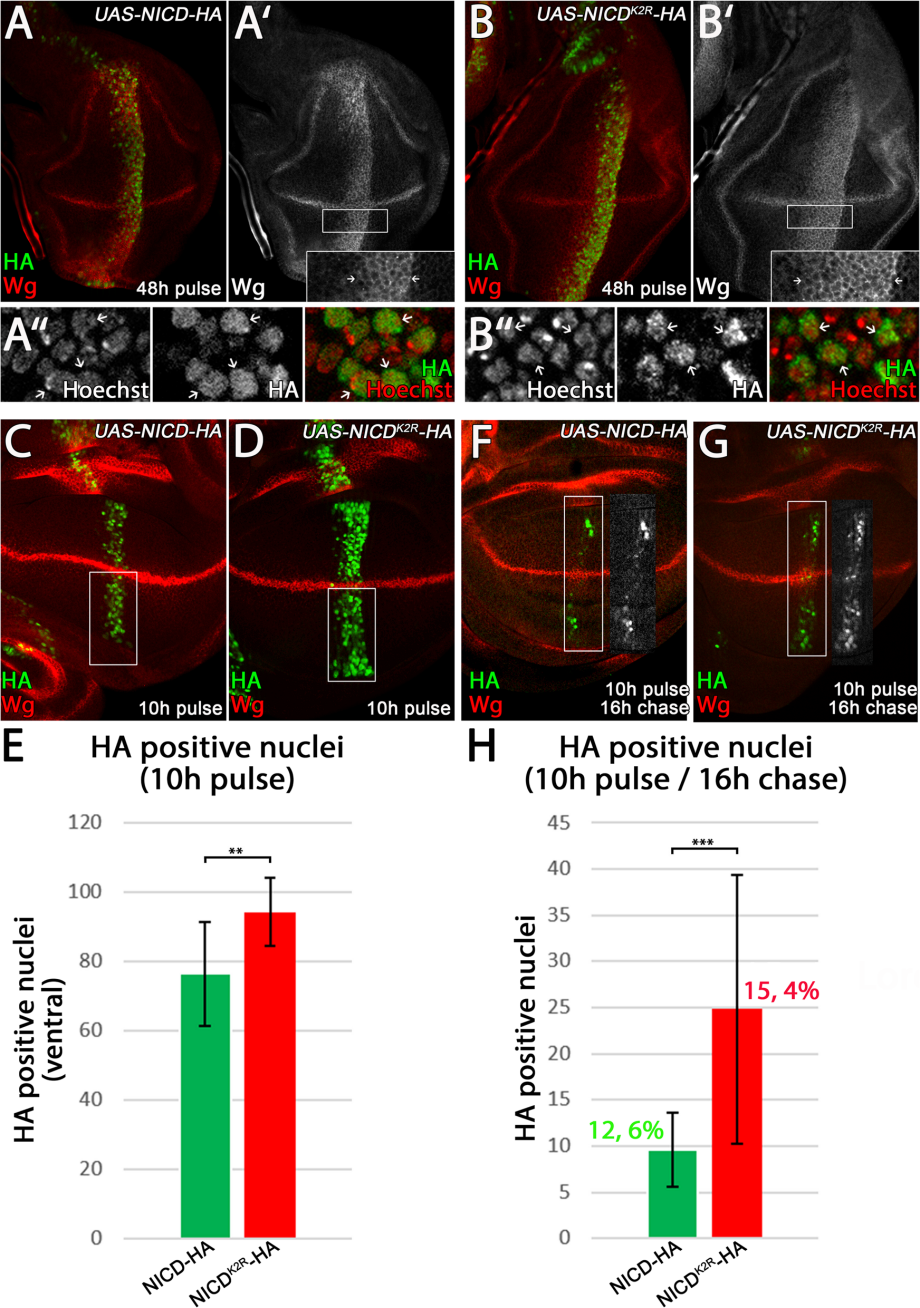
Nuclear degradation of NICD^K2R^-HA is reduced. **A–B”** Expression of NICD-HA and NICD^K2R^-HA in late third instar wing imaginal discs for 48 h under control of *ptc-GAL4 tub-GAL80* ^*ts*^. **A**, **A’**, **B**, **B’** Expression of NICD-HA and NICD^K2R^-HA induced strong ectopic Wg expression along the entire *ptc-Gal4* expression domain in the wing pouch. In contrast to NICD-HA, the Wg expression domain induced by NICD^K2R^-HA was broader (insert in **A’**, **B’**, arrows), indicating that NICD^K2R^-HA has a stronger ability to activate N target genes, even in regions of low expression of the *ptc-Gal4* expression gradient. **A”**, **B”** The magnification highlights the nuclear localisation of the NICD variants. **C–E** Accumulation of NICD-HA and NICD^K2R^-HA in the nucleus after a pulse of expression of only 10 h using *ptc-Gal4* combined with *tub-Gal80* ^*ts*^. **E** The quantification shows that NICD^K2R^-HA accumulated more strongly in the nucleus and in more nuclei than NICD-HA (NICD-HA: *n* = 14 / NICD^K2R^-HA *n* = 12 wing imaginal discs, significance test: Student’s *t* test *p* ≥ 0.05 (n.s.) / *p* ≤ 0.05 (*) / *p* ≤ 0.01 (**) / *p* ≤ 0.001 (***)). **F–H** Pulse-chase assay to analyse the degradation of the NICD constructs in the nucleus. To analyse NICD degradation in the nucleus, NICD-HA and NICD^K2R^-HA expression was pulsed for 10 h and afterwards the larvae were shifted back to a restrictive temperature (18 °C) for 16 h (chase). The remaining HA-positive nuclei in the wing pouch were counted. **H** The quantification shows that significantly more nuclei remained positive for NICD^K2R^-HA than for NICD-HA, suggesting that the degradation of NICD^K2R^-HA in the nucleus was significantly reduced compared to NICD-HA (NICD-HA: *n* = 17 / NICD^K2R^-HA: *n* = 20, significance test: Student’s *t* test *p* ≥ 0.05 (n.s.) / *p* ≤ 0.05 (*) / *p* ≤ 0.01 (**) / *p* ≤ 0.001 (***)). It revealed that after the chase a higher percentage of nuclei remain positive in the case of NICD^K2R^-HA compared to NICD-HA (15.4% vs 12.6%, respectively)

Degradation of NICD in the nucleus is essential for the correct timing of expression of N target genes. It was previously shown that in mammals, NICD is phosphorylated, ubiquitinated, and subsequently degraded by the proteasome [38–40]. Our Western blot analysis indicates that N^K2R^-HA expression generates a stronger band for the cleaved NICD compared to N-HA (Fig. 1E). One reason for this stronger band is its activation in ligand-independent manner in addition to the ligand-dependent one. In order to investigate whether reduced degradation of the cleaved nuclear NICD of N^K2R^-HA might also contribute to the stronger band, we expressed the activated forms of the Notch variants comprising only the ICD, termed NICD-HA and NICD^K2R^-HA. Continuous expression of both of these activated forms resulted in lethality before the third larval instar stage. Therefore, using TARGET, the expression of NICD-HA and NICD^K2R^-HA was restricted to 48 h. *ptc-*GAL4 is expressed in a gradient that increases towards the anterior/posterior (A/P) compartment boundary. This gradient allowed us to analyse the strength of ectopic N signalling by measuring the broadness of the stripe of ectopic expression of the target gene Wg in the anterior direction of lower expression. Both NICD-HA and NICD^K2R^-HA induced strong ectopic expression of Wg and were exclusively localised in the nucleus (Fig. 3A–B’). The ectopic band of Wg expression induced by NICD^K2R^-HA was broader compared to that induced by NICD-HA, indicating that NICD^K2R^-HA has a stronger ability to activate the N target genes also in regions of low expression (Fig. 3A–B’, arrows in magnification). To analyse whether this stronger activation is caused by an increased stability of NICD^K2R^-HA in the nucleus, we performed a pulse-chase experiment in wing imaginal discs and counted the HA-positive nuclei after a chase interval. Upon a pulse of 10 h expression, we detected a significantly higher number of HA-positive nuclei for NICD^K2R^-HA positive than for NICD-HA (average of 94.2 vs 76.3; Fig. 3C, D, quantification in E), suggesting a higher stability of NICD^K2R^-HA. Moreover, upon a subsequent chase of 16 h, a higher percentage of HA-positive nuclei were present for NK2R-HA than N-HA (15.4% vs 12.6%, respectively), supporting a delayed degradation of NICD^K2R^-HA (Fig. 3F, G, quantification in H). Hence, the results indicate that the Ks in the ICD are also important for the proper degradation of the activated form of Notch.

The higher stability of NICD^K2R^-HA probably contributes to the strong ectopic activation of N signalling by N^K2R^-HA, because it leads to a longer maintenance of the activating Su(H) transcription complex. It also contributes to the increase of the NICD band seen in the Western blot for N^K2R^-HA. Nevertheless, the results also indicate that NICD^K2R^-HA is degraded. Hence, also ubi-independent mechanisms contribute to the degradation of NICD in the nucleus.

Altogether, the presented results suggest that the strong ectopic activation of N^K2R^-HA is triggered by two mechanisms: its uncontrolled ectopic activation in a ligand- and Kuz-independent manner and the delayed degradation of the released NICD^K2R^-HA in the nucleus. In addition, similar to N-HA, it can be activated by the ligands.

### N^K2R^-HA is endocytosed and transported through the endosomal pathway

**Fig. 4:**
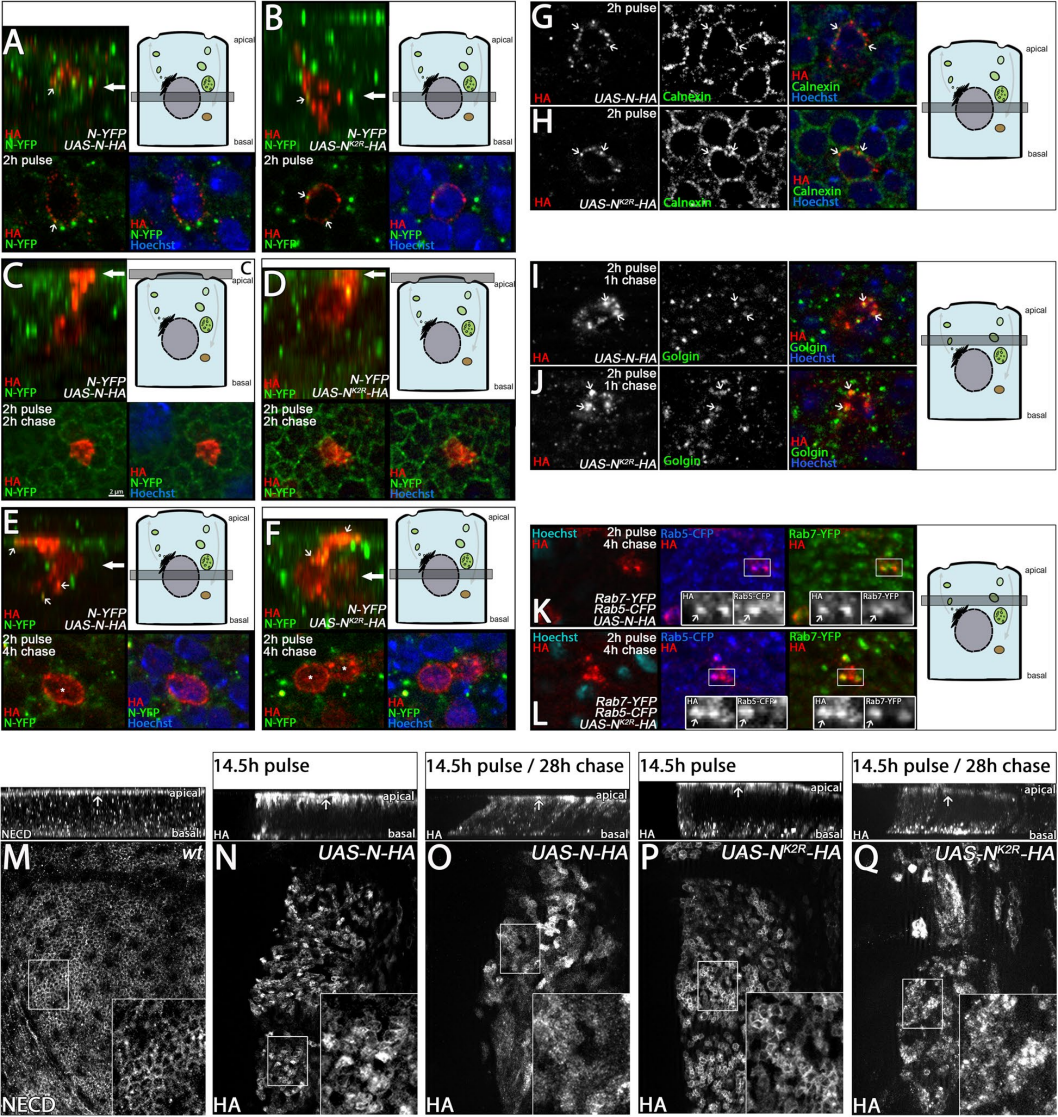
Trafficking of N-HA and N^K2R^-HA through the secretory and endosomal pathway. **A–L** A pulse-chase assay to analyse the subcellular localisation of N-HA and N^K2R^-HA. The pulse of N-HA and N^K2R^-HA expression was limited to 2 h. Following to the pulse, the larvae were shifted back to restrictive 18 °C for different chase intervals. The localisation of the constructs was revealed by HA antibody staining. **A–F** Analysis of N-HA and N^K2R^-HA localisation compared to endogenous N (YFP-N, gene trap). Each box shows a Z-projection (upper panel) and the focal plane (lower panels). The schematic cell and the arrow in the Z-projection shows the focal plane used in each box. Co-localisation of the transgenic constructs and YFP-N is highlighted by small arrows in the corresponding lower panels. **A, B** After a pulse of 2 h, N-HA and N^K2R^-HA were localised in dot-like structures adjacent to the nucleus that in rare cases co-localised with YFP-N. **C, D** After a subsequent chase of 2 h, N-HA and N^K2R^-HA were both localised throughout the apical plasma membrane, while YFP-N was mainly localised to the subapical membrane region, indicated by the honeycomb pattern. **E, F** After a 4 h chase, N-HA and N^K2R^-HA were strongly localised to the nucleus, throughout the apical plasma membrane and in vesicles that co-localise with YFP-N. **G, H** Localisation of N-HA and N^K2R^-HA to the ER. The ER was indicated by Calnexin antibody staining. After the 2-h pulse, the N-HA and N^K2R^-HA-positive dot-like structures adjacent to the nucleus are positive for the ER marker Calnexin (arrows), indicating the early phase of synthesis of the constructs in the ER. **I, J** After 1 h chase, the variants co-localised with the Golgi marker Golgin in the cytosol apical to the nucleus (arrows), indicating that both constructs were in the secretory pathway on their way to the apical plasma membrane via maturation in the Golgi. **K, L** The N-HA and N^K2R^-HA-positive vesicles seen after the 4-h chase were positive for Rab5-CFP and Rab7-YFP (arrows and magnification), indicating that both receptors entered the endosomal pathway. **M–Q** Endocytosis of N-HA and N^K2R^-HA from the apical plasma membrane. To analyse the endocytosis of N-HA and N^K2R^-HA from the apical plasma membrane, the expression of the constructs was increased to a 14.5-h pulse followed by a chase for 28 h. All boxes show a Z-projection (upper panel) and a magnification of the apical focal plane (insert). Areas of magnification are highlighted by the white boxes. **M** Endogenous N was predominantly localised in the apical plasma membrane (honeycomb pattern, insert) and in vesicles throughout the whole A/B axis (z-section in upper panel). The arrow in the z-sections highlights the apical side of the epithelium. **N** After 14.5-h pulse of expression, N-HA localised at the apical plasma membrane (insert), similar to endogenous N. **O** After a subsequent 28-h chase, N-HA levels in the apical plasma membrane were reduced (insert), while it accumulated in intracellular vesicles (z-section in upper panel). **P** Like endogenous N and N-HA, N^K2R^-HA was localised to the apical plasma membrane after 14.5-h pulse of expression (insert). In contrast to N-HA, N^K2R^-HA accumulated also in vesicles at the basal region of the cell (z-section in upper panel). **Q** Similar to N-HA, the levels of N^K2R^-HA in the apical membrane were reduced after a 28-h chase (insert), while the accumulation in basal vesicles continued (z-section in upper panel)

Activation of N depends on endocytosis and endosomal transport [10]. Ubi plays an important role in these processes. Therefore, we were curious whether the replacement of Ks has an impact on the endosomal trafficking of N^K2R^-HA. To monitor the endosomal route of the N-variants, we established a pulse-chase assay in wing imaginal discs using TARGET. In these experiments, we compared the expressed variants to endogenous YFP-tagged N (gene trap, from here on referred to as endogenous N). We found that a pulse of only 2 h expression induced by *hh-*GAL4 *tub*-Gal80^ts^ elicited expression of the variants only in single, well-separated cells in the posterior compartment, enabling the easy tracing of their intracellular route. After the 2-h pulse, N^K2R^-HA and N-HA localised in punctae around the nucleus (Fig. 4A, B, arrow). These punctae overlapped with the ER marker Calnexin (Fig. 4G, H, arrows), indicating that after 2 h pulse N^K2R^-HA and N-HA were just synthesised. After 1 h chase, the localisation of N^K2R^-HA and N-HA also overlapped with the Golgi marker Golgin84, indicating that a fraction was on its anterograde way to the plasma membrane in exosomes (Fig. 4I, J, arrows). After a 2-h chase, both receptors were located throughout the apical plasma membrane. In contrast, endogenous N is mainly localised to the subapical membrane region, indicated by the honeycomb pattern seen in the apical optical section of the epithelium [41, 42] (Fig. 4C, D). Note, that the newly synthesised N-HA and N^K2R^-HA were present only in the compartments of the anterograde transport system located in the apical half of the columnar epithelial cell, indicating a directional transport of the N-variants from the ER to their apical destination. After 4 h of chase, both variants were also located in early (Rab5-CFP positive) and maturing (Rab7-YFP positive) endosomes (Fig. 4K, L, arrows in inserts). Hence, they were endocytosed. Interestingly, at this time point, the HA signal of both N-variants was observed also in the nucleus, suggesting that the ICD of a fraction of each variant was released and transported into the nucleus (Fig. 4E, F, asterisks in lower panel). Despite the activation of the variants, activation of N target genes was not observed at this time point. Probably, it was below the level of detection. Taken together, the results show that the replacement of the Ks has no qualitative impact on receptor synthesis and anterograde delivery of N to the apical surface. Moreover, endocytosis of N and trafficking through the endosomal pathway is possible without Ks and ubi. However, while we could rule out gross defects in endocytosis of N^K2R^-HA, we cannot rule out differences in its efficiency. The localisation of N^K2R^-HA and N-HA inside the nucleus suggests that their ICDs are cleaved off and translocated into the nucleus.

To get further insight into the apical localisation and endocytosis of the N variants, the pulse of expression was increased to 14.5 h. In contrast to the 2-h pulse, the longer expression resulted in an accumulation of N^K2R^-HA and N-HA at the subapical membrane where also endogenous N is located (Fig. 4M, N, P). In addition, endogenous N, N^K2R^-HA, and N-HA were localised in vesicles along the apico-basal axis of the disc cells. In contrast to N-HA, vesicles containing N^K2R^-HA accumulated in the basal half of the cells and appeared to be increased (Fig. 4P, z-section, compare with N). If the variants were chased for 28 h after the 14 h pulse, the amount of both variants at the subapical domain decreased, while N positive vesicles were still visible along the apico-basal axis of the cells. Note, that the strong accumulation of N^K2R^-HA-positive vesicles in the basal half of the cell remained (Fig. 4Q, z-section, compare with that of O).

Taken together, these experiments show that the Ks of the ICD of Notch are not essential for anterograde transport of the receptor to the apical membrane, nor for its endocytosis from the apical membrane. The prevailing difference between N-HA and N^K2R^-HA was the accumulation of N^K2R^-HA in basally located enlarged vesicles after endocytosis.

### N^K2R^-HA accumulates in the LM of abnormal endosomes

**Fig. 5:**
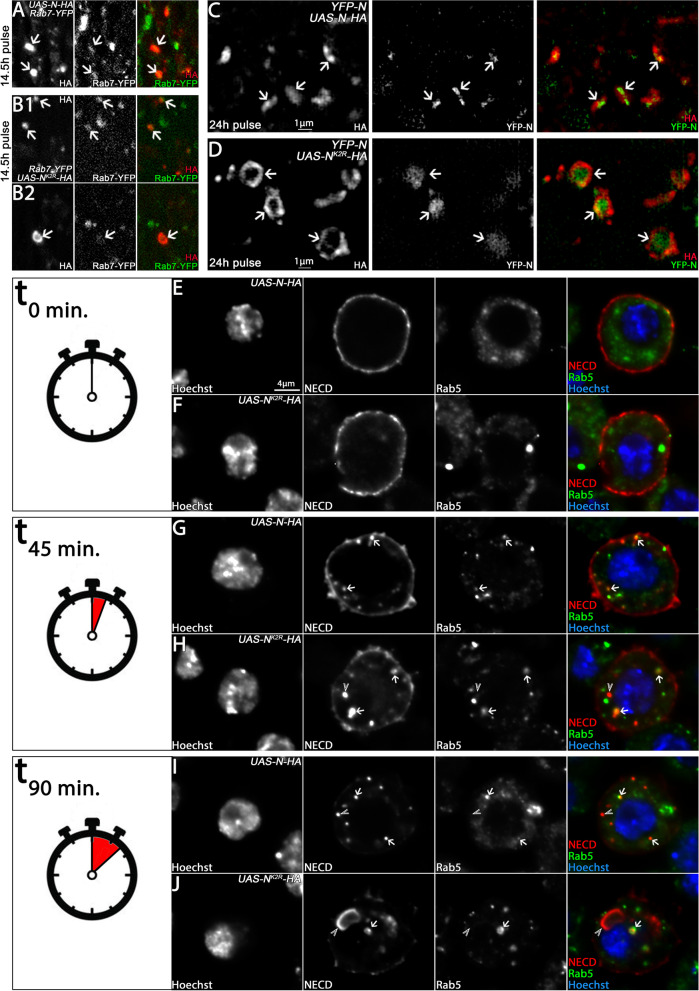
N^K2R^-HA accumulates at the limiting membrane of abnormal endosomes. **A–B2** Endosomal localisation of N-HA and N^K2R^-HA after 14.5 h expression. Endosomes were indicated by tub-Rab7-YFP. The arrows point some of the analysed endosomes. **A** N-HA localised on Rab7-positive endosomes. **B1** Similar to N-HA, apical N^K2R^-HA localised on Rab7-positive endosomes. **B2** In contrast to N-HA, basally located N^K2R^-HA-positive vesicles were enlarged and were hardly positive for Rab7. **C**, **D** Analysis of basal N-HA and N^K2R^-HA vesicles with structured illumination microscopy (SIM). N-HA and N^K2R^-HA were expressed for 24 h. **C** N-HA clearly co-localised with endogenous YFP-N (arrows) in the basal endosomes. **D** In contrast, N^K2R^-HA was restricted to the limiting membrane of the enlarged vesicles. Note, that the endogenous YFP-N was clearly localised in the vesical lumen (the area framed by the N^K2R^-HA signal), indicating that the ESCRT-mediated ILV formation occurred, but N^K2R^-HA was not incorporated into the ILVs (arrows). **E–J** Uptake-assay of N-HA and N^K2R^-HA in S2 cells. N-HA and N^K2R^-HA were expressed for 24 h under control of *pMT-GAL4*. After expression, the cells were shifted to 4 °C and incubated with NECD antibody, which exclusively detects the N extracellular domain on the cell surface. Afterwards, the cells were shifted back to 25 °C to re-initiate endocytosis and fixed and stained with secondary antibody after 0’, 45’ and 90’ of re-initiated endocytosis. Endosomes were revealed by anti Rab5 antibody staining. Co-localisation of N-HA and N^K2R^-HA with Rab5-positive endosomes is highlighted by the arrows. The arrowheads show N-HA or N^K2R^-HA vesicles that do not co-localise with Rab5. **E**, **F** At *t* = 0, N-HA and NK2R-HA were predominantly localised at the plasma membrane. **G**, **H** At *t* = 45 min, N-HA and N^K2R^-HA were endocytosed and located in Rab5-positive endosomes. **I**, **J** At *t* = 90 min, N-HA and N^K2R^-HA were nearly completely removed from the plasma membrane, indicated by the loss of membrane staining. **J** In contrast to N-HA, N^K2R^-HA was also localised at the limiting membrane of enlarged endosomes that were negative for Rab5, indicating that abnormal N^K2R^-HA-positive endosomes had formed

To determine the nature of the accumulating N^K2R^-HA-positive vesicles in the basal region, we used the ME marker Rab7-YFP. This analysis showed that N-HA and N^K2R^-HA are localised mainly in Rab7-positive MEs (Fig. 5A, B1, arrows). Note, that the enlarged vesicles at the basal compartment were only weakly positive for Rab7-YFP or not positive at all (Fig. 5B2, arrows). Furthermore, N^K2R^-HA was distributed in a ring-shaped domain at the circumference of these vesicles, suggesting that it accumulated in their LMs, instead of being transported into the lumen. The number of such enlarged basally located vesicles increased dramatically if N^K2R^-HA was expressed for longer periods (Fig. S2). Due to the loss of endosomal markers, we called these vesicles “ghost” vesicles (GVs). Note, that we did not observe these GVs upon expression of N-HA (Fig. 5C, D).

To further determine the localisation of N^K2R^-HA in the GVs, we turned to super-resolution microscopy (SIM). This analysis revealed a clear difference in the localisation of N^K2R^-HA compared to endogenous N and N-HA on GVs. N-HA was distributed evenly throughout the vesicle perimeter and strongly overlapped with endogenous Notch (Fig. 5C, arrows). In contrast, N^K2R^-HA was restricted to the LM, while endogenous N was predominantly localised in the lumen of GVs (Fig. 5D, arrows). Thus, the replacement of intracellular Ks appears to prevent the internalisation of N into the lumen of MEs and the development of GVs.

To further understand the genesis of the GVs, we established an uptake-assay in S2 cells (Fig. 5E–J). The fact that S2 cells do not express N allowed us to exclusively chase the transfected constructs. In this assay, we expressed N^K2R^-HA or N-HA under control of the *pMT*-GAL4 driver for 24 h. To exclusively label the surface fraction of N-HA and N^K2R^-HA, we blocked endocytosis by a shift of the cells to 4 °C during the incubation with an antibody directed to the extracellular domain of Notch (NECD). The antibody-labelled fraction of N on the cell surface can then be chased through the endosomal pathway upon endocytosis initiated by shift of the temperature to 25 °C by subsequent antibody staining. As expected, N^K2R^-HA and N-HA were detected exclusively at the plasma membrane at 0’ of chase (Fig. 5E, F). After a chase of 45’, a fraction of N^K2R^-HA and N-HA localised also to early Rab5-positive endosomes (Fig. 5G, H, arrows). After 90’, the labelled receptors were nearly completely endocytosed. Some of the formed endosomes were still positive for Rab5 (Fig. 5I, J, arrows). Interestingly, cells that expressed N^K2R^-HA formed Rab5-negative enlarged vesicles that cannot be detected in the case of N-HA. They resembled the GVs seen in disc cells. It appears that the expression of N^K2R^-HA in cells results in the formation of initially Rab5-positive endosomes that gradually lose their endosomal identity during maturation and enlarge dramatically.

### Dx and Su(dx) induce ubi-independent endocytosis of N

We here observed that endocytosis of N^K2R^-HA from the apical membrane of the imaginal disc epithelium can be mediated in an ubi-independent manner. Since Dx and Su(dx) are E3 ubiquitin ligases that contribute to the regulation of trafficking and signalling of N, we wondered whether they can regulate endosomal trafficking of N^K2R^-HA. Previous data showed that overexpression of a Su(dx) variant without the ubi-mediating HECT domain still induces N endocytosis [19, 20]. Moreover, a Dx variant where the RING domain was replaced by a dimerising GST domain is still functional [18]. These findings suggest that Dx and Su(dx) can induce N endocytosis in an ubi-independent manner. To confirm this notion, we analysed the impact on co-expression of Dx and Su(dx) on trafficking and signalling of the N-variants.

**Fig. 6:**
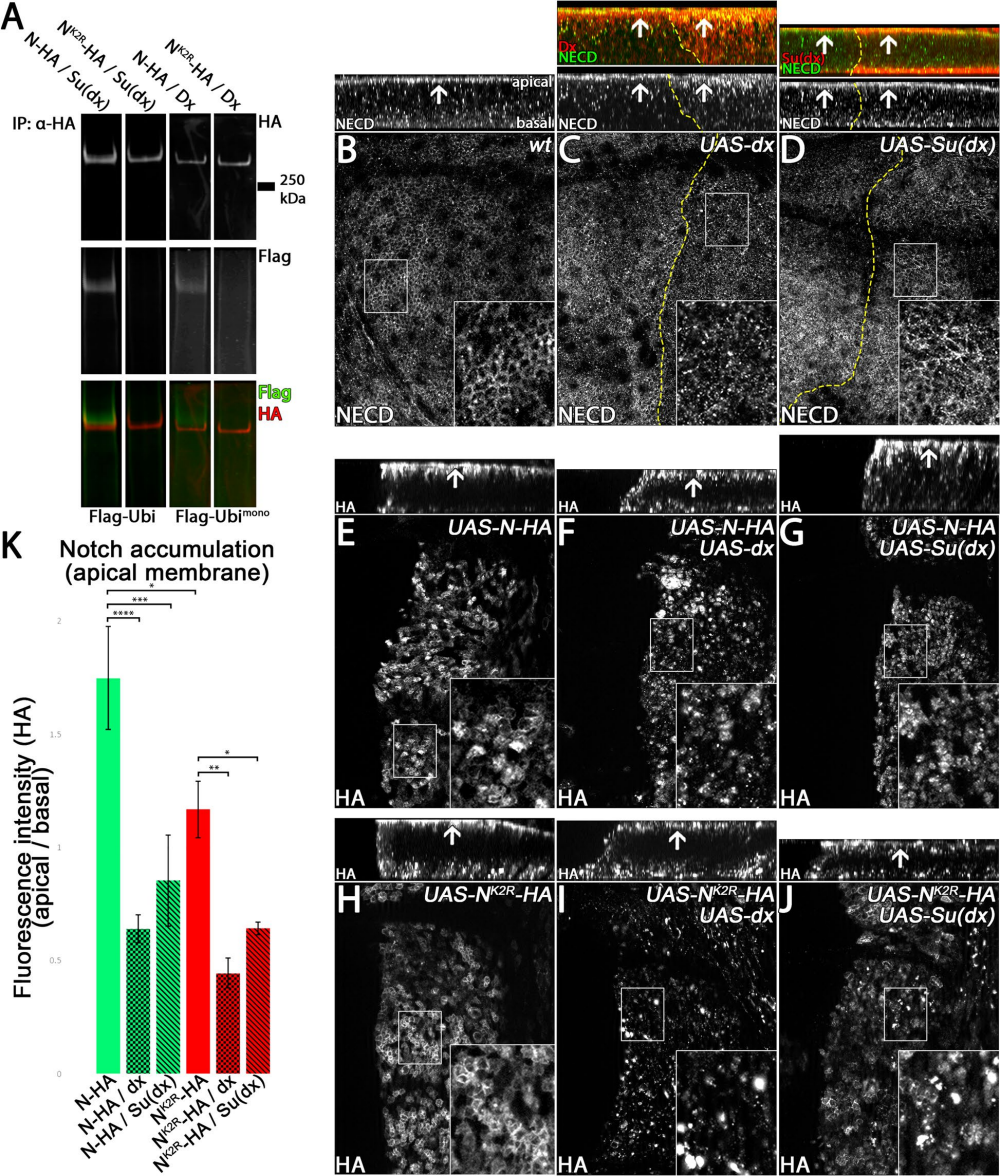
Dx and Su(dx) induce ubiquitination-independent endocytosis of N. **A** Ubiquitination-assay of N-HA and N^K2R^-HA. The variants were co-expressed with Dx or Su(dx) and Flag-tagged Ubiquitin (Flag-Ubi) in S2 cells and then immuno-precipitated with an HA antibody and analysed by Western blotting. N-HA and N^K2R^-HA bands were revealed by HA antibody staining. Ubiquitin was revealed by anti-Flag antibody staining. The results show that N-HA, but not N^K2R^-HA, was ubiquitinated by Dx, as well as Su(dx), indicating that Dx and Su(dx) ubiquitinate the ICD of N on Ks. **B–J** Dx and Su(dx) mediated endocytosis of N, N-HA and N^K2R^-HA. N-HA and N^K2R^-HA were co-expressed with Dx or Su(dx) for 14.5 h under control of *hh-GAL4 tub-GAL80ts*. Each box shows a Z-projection of (upper panel) and a focal plane at the apical plasma membrane (insert) of late third instar larvae wing imaginal discs. **B–D** As a control, endogenous YFP-N was monitored in wild-type discs and disc overexpression of Dx (**C**) and Su(dx) (**D**). In wild-type cells, N localised at the subapical membrane domain, generating the characteristic honeycomb pattern observed in the corresponding optical plane. **C** Expression of Dx induced strong endocytosis of N from the apical membrane, indicated by the loss of the apical honeycomb pattern (insert) and increased vesicle formation (upper panel). **D** In contrast to Dx, Su(dx) had no obvious effect on the subcellular localisation of N. **E–G** Co-expression of N-HA with Dx or Su(dx). N-HA was predominantly localised at the apical plasma membrane if expressed alone. **F**, **G** Co-expression of N-HA with Dx, or Su(dx), resulted in strong decrease of the apical N-HA membrane fraction, indicating that Dx and Su(dx) can induce endocytosis of N-HA from the plasma membrane. **H** Likewise, N^K2R^-HA strongly accumulated at the apical plasma membrane upon its expression. **I**, **J** Like in the case of N-HA, co-expression with Dx or Su(dx) induced a strong decrease of the N^K2R^-HA membrane fraction, indicating that Dx and Su(dx) can induce endocytosis of N^K2R^-HA in a ubiquitin-independent manner. **K** Quantification of Dx and Su(dx) induced endocytosis of N-HA and N^K2R^-HA. For quantification, the HA fluorescence intensity of the apical plasma membrane was compared to that over the apical-basal axis without the plasma membrane (for each genotype *n* = 3 wing imaginal discs were analysed, significance test: one-way ANOVA *p* ≥ 0.05 (n.s.) / *p* ≤ 0.05 (*) / *p* ≤ 0.01 (**) / *p* ≤ 0.001 (***) / *p* ≤ 0.0001 (****)). Dx and Su(dx) can significantly reduce the membrane fraction of N-HA and N^K2R^-HA, indicating that Dx and Su(dx) can induce endocytosis of N-HA and N^K2R^-HA. Compared to Dx, the ability of Su(dx) to induce endocytosis of N-HA and N^K2R^-HA was reduced

We first asked whether Dx or Su(dx) can ubiquitinate N^K2R^-HA in an ubi-assay in S2 cells (Fig. 6A). In this assay, N-HA or N^K2R^-HA were co-expressed with Dx or Su(dx) and Flag-tagged ubiquitin (Flag-Ubi). N-HA and N^K2R^-HA were precipitated and analysed by western blot analysis. Ubi was revealed by anti-Flag antibody staining. The assay showed that only N-HA, but not N^K2R^-HA, was ubiquitinated by Dx and Su(dx) (for whole cell extracts and negative control see Fig. S3). Hence, ubi of N by Dx and Su(dx) appears at the Ks of the ICD of N.

Next, we analysed the impact of over-expression of Dx or Su(dx) on the localisation of endogenous N (Fig. 6B–J). To do so, Dx or Su(dx) were expressed for 14.5 h in wing imaginal discs and then the apical localisation of endogenous N monitored. We found that the expression of Dx increased endocytosis of N from the apical membrane (Fig. 6C). In contrast, expression of Su(dx) had no obvious impact on apical N localisation (Fig. 6D). Since it was shown that Su(dx) is more active at higher temperature [19], we repeated the experiment at 29 °C. But even in this situation, we failed to detect a clear effect (Fig. S4A-B’). Thus, Dx appears to have the predominant role in initiating endocytosis of endogenous N.

In agreement to what we observed with endogenous N, the co-expression of N-HA with Dx increased N-HA endocytosis from the apical membrane (Fig. 6E, F, quantification in K). In contrast to endogenous N, Su(dx) expression increased endocytosis of co-expressed N-HA (Fig. 6E, G, K). Importantly, endocytosis of N^K2R^-HA was also increased by either Dx or Su(dx) co-expression (Fig. 6H. I, quantification in J, K). Similar to N-HA, endocytosis of N^K2R^-HA mediated by Dx was stronger compared to Su(dx) (quantification in Fig. 6K).

Altogether, these results confirm that Dx can induce endocytosis of Notch in an ubi-independent manner. Moreover, the observed increased endocytosis of N-HA induced by Su(dx) suggests that Su(dx) controls endocytosis when N is expressed at high levels.

### Dx and Su(dx) regulate the endosomal localisation and activation of N-HA and N^K2R^-HA

Since Dx and Su(dx) can induce ubi-independent endocytosis of N^K2R^-HA, we next asked if they can influence its activity. Over-expression of Dx induced strong accumulation of N at the limiting endosomal membrane and also ectopic ligand-independent N signalling. In contrast, Su(dx) increased the internalisation of N into the endosomal lumen and thereby suppresses ectopic N signalling [19–21].

**Fig. 7:**
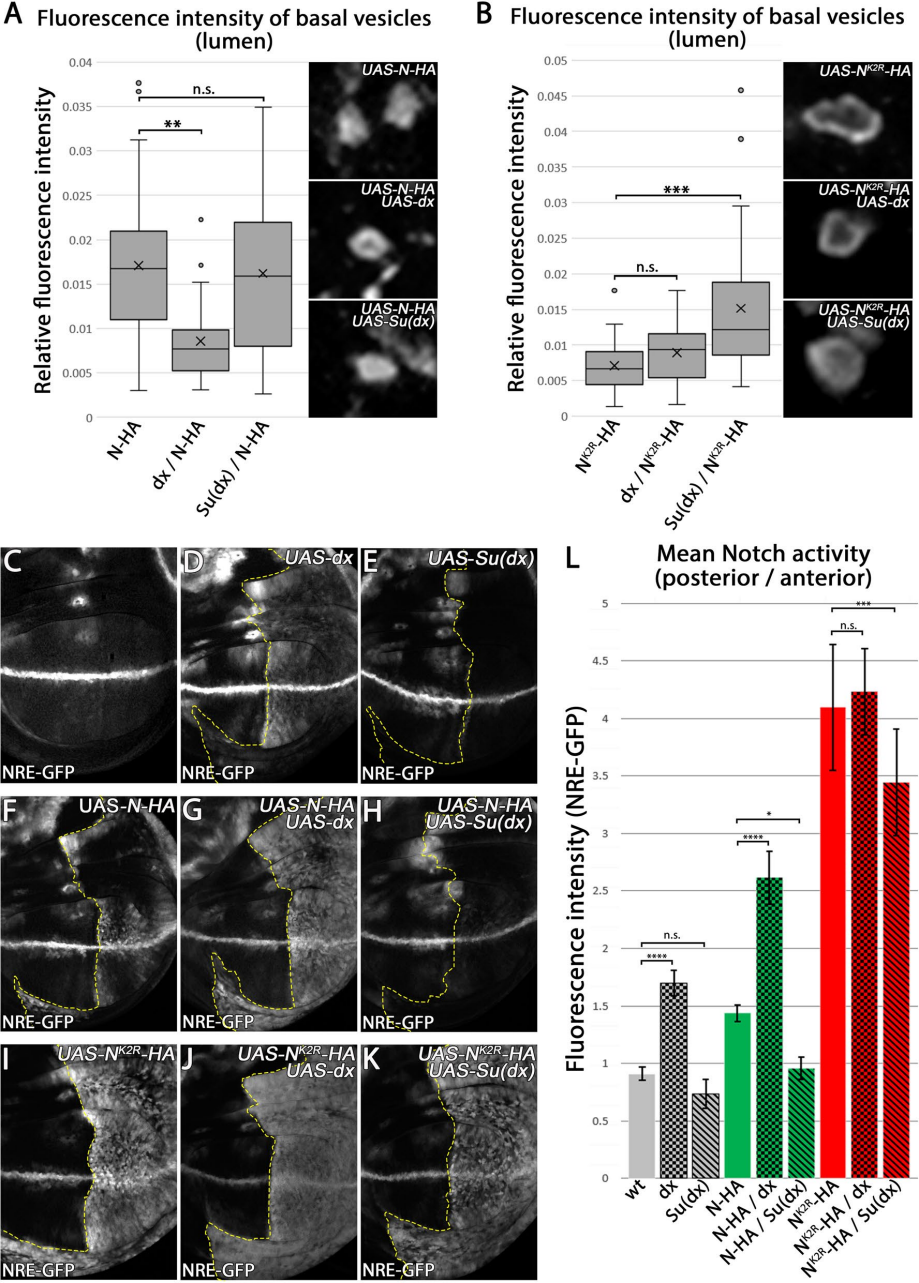
Dx and Su(dx) regulate the endosomal localisation and activation of N-HA and N^K2R^-HA. **A, B** Quantification of the fluorescence intensity of the lumen of basal N-HA and N^K2R^-HA-positive vesicles. The HA fluorescence intensity along the equator of the vesicle was measured. The difference of the lowest intensity of each vesicle lumen and the highest intensity of each vesicle limiting membrane was determined. To show the fraction of N-HA and N^K2R^-HA inside the lumen, the calculated fluorescence difference was plotted reciprocally in a box plot. **A** While Dx induced a decrease in accumulation of N-HA in the lumen of the vesicles, Su(dx) had no detectable effect on N-HA internalisation (for each genotype *n* = 30 vesicles of the 3 wing imaginal discs were analysed, significance test: one-way ANOVA *p* ≥ 0.05 (n.s.) / *p* ≤ 0.05 (*) / *p* ≤ 0.01 (**) / *p* ≤ 0.001 (***) / *p* ≤ 0.0001 (****)). **B** Dx had no further effect on NK2R-HA accumulation at the limiting membrane of basal vesicles. In contrast, Su(dx) increased the internalisation of N^K2R^-HA into the vesicle lumen, indicating that Su(dx) induces ubiquitination-independent internalisation of N^K2R^-HA (for each genotype *n* = 30 vesicles of the 3 wing imaginal discs were analysed, significance test: one-way ANOVA *p* ≥ 0.05 (n.s.) / *p* ≤ 0.05 (*) / *p* ≤ 0.01 (**) / *p* ≤ 0.001 (***) / *p* ≤ 0.0001 (****)). **C–K** Activation of the N pathway by Dx and Su(dx). To analyse the impact of Dx and Su(dx) on N signalling, they were expressed alone or together with N-HA, N^K2R^-HA for 14.5 h under control of *hh-GAL4 tubGAL80* ^*ts*^ in the posterior compartment of the wing imaginal disc. N signalling was revealed by NRE-GFP. The dashed yellow line highlights the anterior boundary of the expression domain. Expression by hh-Gal4 is restricted to the right of the line. **L** For quantification of N signalling shown in **C–K**, the fluorescence intensity of NRE-GFP was measured and normalised to that of the anterior control compartment (wt: *n* = 9 / dx: *n* = 10 / Su(dx) *n* = 6 / N-HA: *n* = 7 / N^K2R^-HA: *n* = 11 / N-HA dx: *n* = 8 / N-HA Su(dx): *n* = 7 / N^K2R^-HA dx: *n* = 13 / N^K2R^-HA Su(dx) *n* = 9 wing imaginal discs, significance test: one-way ANOVA *p* ≥ 0.05 (n.s.) / *p* ≤ 0.05 (*) / *p* ≤ 0.01 (**) / *p* ≤ 0.001 (***) / *p* ≤ 0.0001 (****)). Dx induced significantly increased signalling of endogenous N, while in the case of Su(dx) only a slight tendency to reduce endogenous N signalling was observed. N-HA-mediated signalling was increased by Dx and decreased by Su(dx). In contrast, N^K2R^-HA-mediated signalling could not be increased by Dx, but was decreased by Su(dx), indicating that Su(dx) can decrease N^K2R^-HA-mediated N signalling by a ubiquitination-independent mechanism

In order to investigate the distribution of the transgenic N constructs in MEs, we measured the ratio between the amount of the N variants at the LM and the lumen of MEs (Fig. 7). In contrast to Su(dx), Dx over-expression induced accumulation of endogenous N at MEs that, in rare cases, showed a slight lumen in cross-sections (Fig. S4C, D). We confirmed that the over-expression of Dx induced ectopic N signalling, while over-expression of Su(dx) tended to suppress N signalling (Fig. 7C–E, L / Fig S4 A’) [22, 43]. Co-expression of N-HA with Dx resulted in a decreased fraction of N-HA inside the endosome lumen, combined with increased N signalling (Fig. 7A, F, G). In contrast, co-expression with Su(dx) caused no significant difference in the ratio of N-HA at membrane and lumen of endosomes compared to N-HA single expression, but suppressed ectopic N signalling (Fig. 7A, F–H, L).

The results further support the finding of Shimizu et al. (2014) that Dx prevents internalisation of N into the lumen of endosomes and indicates that the fraction of N in the LM induces N signalling. Contrary to the expectation, Su(dx), however, showed no increased internalisation of N-HA into the vesicular lumen, but suppressed the ectopic N signalling mediated by N-HA. This might be due to the fact that N-HA, if expressed alone, already strongly localises to the vesicular lumen and the possible additional internalisation mediated by Su(dx) might be below the level of detection. As shown above, N^K2R^-HA expression leads to formation of GVs with strong accumulation of N^K2R^-HA at the LM. Co-expression of N^K2R^-HA with Dx does not alter this phenotype and does not affect the induced strong ectopic N signalling (Fig. 7B, I, J).

### A di-leucine motif as an alternative endocytosis signal

**Fig. 8:**
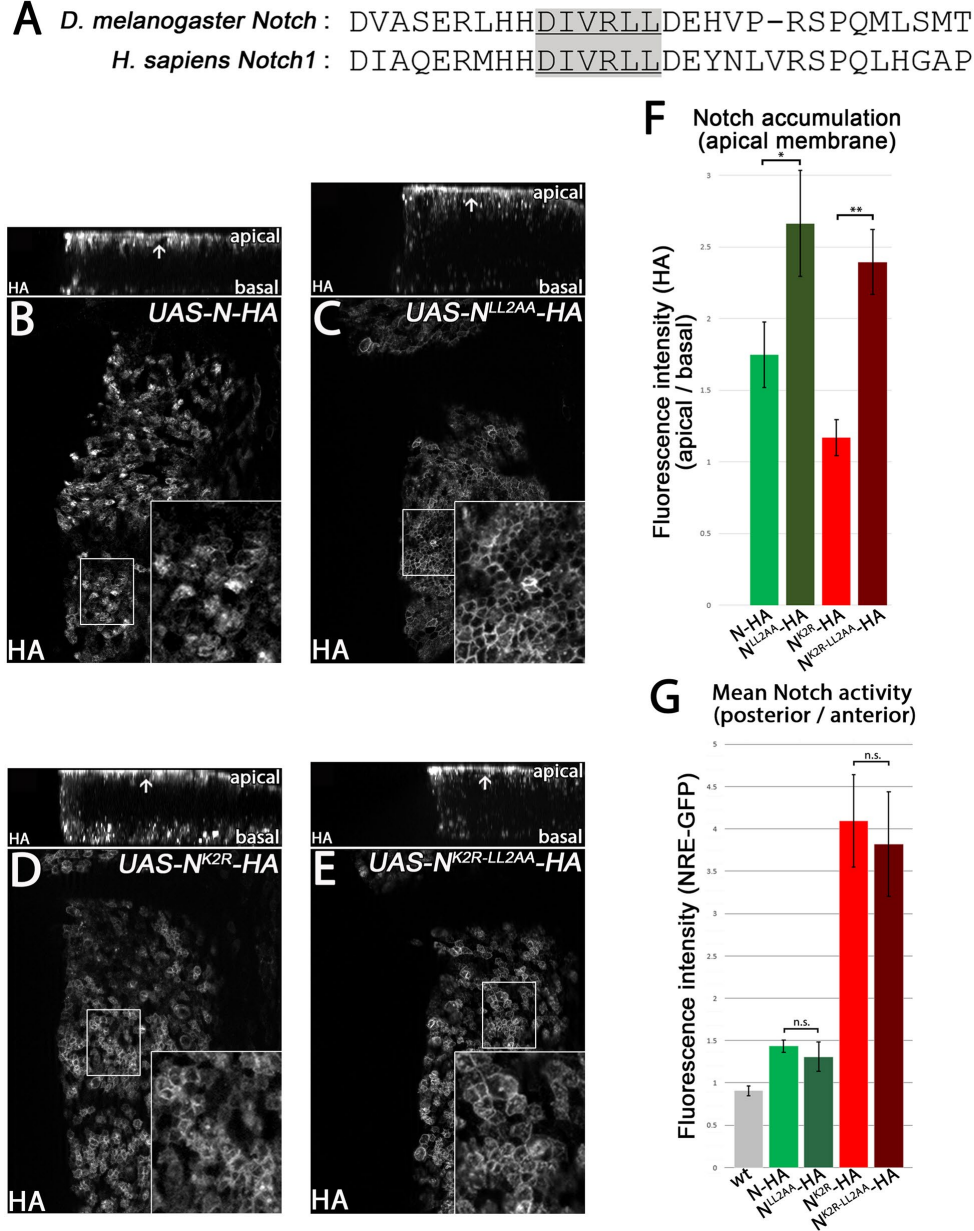
A di-leucine motif as an alternative endocytosis signal in Notch. **A** *Drosophila* N and human N1 share a conserved di-leucine motif in its intracellular domain. **B–E** Endocytosis of N-HA, N^LL2AA^-HA, N^K2R^-HA and N^K2R-LL2AA^-HA. The N constructs were expressed for 14.5 h under control of *hh-GAL4 tub-GAL80* ^*ts*^. Each picture shows a Z-projection of the A/B axis (upper panel) and a focal plane of the apical plasma membrane (lower panel). Areas of magnification are highlighted by white boxes. Localisation of the transgenic constructs was indicated by HA antibody staining. Replacement of the di-leucine motif resulted in increased accumulation of N^LL2AA^-HA and N^K2R-LL2AA^-HA in the apical plasma membrane, indicated by the strong honeycomb structure (lower panels). Despite the increased membrane localisation, N^LL2AA^-HA and N^K2R-LL2AA^-HA were present in vesicles (upper panels). **F** To quantify the impact of the LL2AA mutant on endocytosis, the HA fluorescence intensity of the apical plasma membrane was related to the total HA fluorescence intensity area along the A/B axis, without the plasma membrane signal (for each genotype *n* = 3 wing imaginal discs were analysed, significance test: one-way ANOVA *p* ≥ 0.05 (n.s.) / *p* ≤ 0.05 (*) / *p* ≤ 0.01 (**) / *p* ≤ 0.001 (***) / *p* ≤ 0.0001 (****)). The replacement of the di-leucine motif significantly increased the membrane fraction of N-HA, as well as N^K2R^-HA, indicating that the di-leucine motif mediates N endocytosis in a ubi-independent manner. **G** The meaning of the di-leucine motif for the activity of N-HA and N^K2R^-HA. The variants were expressed for 14.5 h under control of *hh-GAL4 tub-GAL80* ^*ts*^. For the quantification the activity, the fluorescence intensity of NRE-GFP in the posterior compartment was normalised to the anterior compartment (wt: *n* = 9 / N-HA: *n* = 7 / N^K2R^-HA: *n* = 11 / N^LL2AA^-HA: *n* = 8 / N^K2R-LL2AA^-HA: *n* = 7 wing imaginal discs, significance test: one-way ANOVA *p* ≥ 0.05 (n.s.) / *p* ≤ 0.05 (*) / *p* ≤ 0.01 (**) / *p* ≤ 0.001 (***) / *p* ≤ 0.0001 (****)). Despite the observed reduced endocytosis, the di-leucine motif had no significant impact on the activity of N-HA and N^K2R^-HA

The results so far show that N^K2R^-HA can be endocytosed in an ubi-independent manner. Moreover, Dx and Su(dx) influence the strength of N endocytosis in an ubi-independent way. Ubi-independent endocytosis is mostly mediated by motifs located in the ICD of cargo proteins, such as tyrosine and di-leucine motifs [44]. Interestingly, *Drosophila* N contains an evolutionary conserved di-leucine (LL) motif whose function was previously analysed for NOTCH1 (Fig. 8A) [45]. In this case, it appears be not necessary for endocytosis, but for the transport of NOTCH1 from early to late endosomes. A version that lacks the motif had reduced signalling abilities.

To analyse the role of the LL motif in N, we replaced the leucines by alanines analogous to Zheng et al. [45] and tested the endocytosis and activity of this variant (N^LL2AA^-HA). Compared to N-HA, N^LL2AA^-HA showed a stronger localisation to the subapical region, indicated by the honeycomb pattern of localisation (Fig. 8B, C). Moreover, fluorescence intensity measurements revealed a significant increase in apical membrane localisation compared to N-HA (Fig. 8F). However, we still detected N^LL2AA^-HA in intracellular vesicles, suggesting that endocytosis was not completely suppressed (Fig. 8C, z-section / Fig. S5 A). Collectively, the results suggest that, in contrast to NOTCH1, the LL motif in N, constitutes a motif for endocytosis rather than for endosomal transport.

To further confirm that N is endocytosed by ubi-dependent and ubi-independent mechanisms, we replaced the LL motif in N^K2R^-HA (N^K2R-LL2AA^-HA). Consistent with N^LL2AA^-HA, the apical fraction of N^K2R-LL2AA^-HA is also increased compared to N^K2R^-HA (Fig. 8D, E, F). Interestingly, endocytosis of N^K2R-LL2AA^-HA is also only reduced, but not abolished, suggesting that endocytosis of N can be induced even by an ubi- and LL motif-independent mechanism (Fig. 8E, z-section / Fig. S5B).

Surprisingly, neither N^LL2AA^-HA nor N^K2R-LL2AA^-HA differ significantly in their activity compared to N-HA or N^K2R^-HA (Fig. 8G). Therefore, the LL motif appears to regulate N endocytosis, but plays a minor role in N signalling.

## Discussion

Here, we investigated the impact of ubi on the activity and endocytic trafficking of the N signalling receptor. For this purpose, we characterised a variant where all Ks in its ICD are replaced by Rs. We found that the Ks are required for incorporation of endocytosed Notch into ILVs to prevent the uncontrolled ligand-independent activation of Notch and to regulate the degradation of its activated ICD. However, despite the existence of several E3 ligases devoted to Notch, the Ks are not essential for its endocytosis. In addition, we could confirm that Dx and Su(dx) can induce endocytosis of N in an ubi-independent manner and that both E3 ligases regulate the amount of N at the LM of MEs.

Given the involvement of E3 ligase-mediated ubi in endocytosis, one expectation at the beginning of our investigation was that N^K2R^-HA might not be endocytosed. However, we could not detect any obvious difference in the efficiency of endocytosis between N-HA and N^K2R^-HA. Moreover, the single-cell pulse-chase experiments indicate that both variants are correctly transported to the apical plasma membrane where they are localised in a domain that overlaps with that of endogenous N. We also found no qualitative differences in the endocytosis of both variants in this assay. While we cannot rule out the existence of small differences, the observations indicate that ubi of the ICD of N is not an essential requirement for endocytosis. A similar conclusion has been drawn for the role of ubi in endocytosis of the Notch ligand Dl [46]. Cargo can also be endocytosed through the presence of sorting motifs in their cytosolic domains [10, 44]. These motifs mediate the direct binding to core components of the endocytic machinery, such as the adapter AP-2. One of these signals is the LL-motif, which is present in all N orthologs and has been shown to be involved in the endocytosis of the EGF-R [47]. The sequence in Notch, DIVRLL, fits well to the consensus sequence D(E)XXXL(L/I), which mediates binding to the endocytic adapter the AP-2 [48]. We here found that it contributes to the endocytosis of N but, like ubi, is not essential. Even if both endocytosis signals, Ks and LL motif, are deleted, Notch is still active and endocytosed. These results indicate that, as in the case of Dl, several mechanisms operate in parallel during endocytosis of N. This is probably an explanation for the very weak mutant phenotypes of E3 ligases that are shown to be involved in N endocytosis, e.g. Dx and Su(dx). It is also a likely explanation for the little effect the blocking of one of the endosomal pathways has on signalling or two of the endosomal processes does not affect signalling in a detectable manner.

A major difference between N-HA and N^K2R^-HA was that, in contrast to N-HA or endogenous N, N^K2R^-HA was not imported from the LM into the lumen of the ME. This import is regulated by the ESCRT machinery via formation of ILVs. To be recognised by the initiating early ESCRT complexes, the cargo must be poly-ubiquitylated in its cytosolic domain [10, 14, 49]. Thus, the likely explanation for the failure of incorporation of N^K2R^-HA into ILVs is that it is not recognised by the early ESCRT complexes. Consequently, it fails to be incorporated into ILVs and accumulates in the LM of the ME. This finding indicates that ubi of N is not essential for endocytosis, but for the incorporation of Notch into ILVs of the ME. The ubi can already take place at the plasma membrane by E3 ligases such as Dx and Su(dx) and then have an impact on the behaviour of N at the endosome.

We found that the longer expression of N^K2R^-HA resulted in the formation of endosomes that gradually loose the endosomal marker Rab7 and become what we called GVs. It has to be pointed out that ILV formation is not disturbed in the GVs, since we found that the endogenous YFP-Notch is localised in the lumen of the GVs. Thus, a lack of ILV formation, which would lead to the remaining of the corresponding membrane part in the limiting membrane, does not contribute to the enlargement of the endosomes/GVs in N^K2R^-HA expressing cells. Rab7 is the central organiser of events on MEs, such as the fusion with the lysosome. Its loss prevents the fusion of the ME with the lysosome and dramatically increases the lifetime of the ME [50]. This increase in lifetime allows more homotypic fusion events to occur and is therefore probably the cause of the increased in size of the MEs/GVs.

How the gradual loss of Rab7 is caused by accumulation of N^K2R^-HA remains to be resolved. However, one can imagine that the accumulation of high amounts of N^K2R^-HA in the LM disturbs the formation of relevant protein complexes and functional domains organised by the Rab proteins, e.g. the Mon1/Ccz1 complex that recruits Rab7 [51]. These disturbances could cause the gradual degeneration of ME into GVs. In accordance with our assumption, a recent paper reports that the accumulation of the Notch receptor is the cause of the dramatic enlargement of MEs in retromer complex mutants [52].

Since N^K2R^-HA is not included in ILVs and accumulates in the LM, one could imagine that if N^K2R^-HA cannot be degraded then it might be recycled. It has been shown that Notch is recycled in a retromer-dependent manner in neuroblasts [52]. We also previously reported that a small fraction of Notch is recycled in a Rme8-dependent manner in the wing imaginal disc [53]. While our experiments were not designed to differences in recycling, we have not noticed strong enhanced recycling with N^K2R^-HA in imaginal discs compared to N-HA. Rather, we observed that N^K2R^-HA accumulates in the limiting membrane of MEs, which as a result gradually become GVs. Enhanced recycling for Notch is also not reported for the case of the loss of function of *hrs*, which encodes an ESCRT-0 component [36, 37, 54]. Moreover, we have recently found that in cells depleted of *shrub* or *vps4* function, which encode central ESCRT-III components, Notch is not recycled in a recognisable enhanced manner, but accumulates in enlarged aberrant MEs where it is activated [35, 55]. In these cases, Notch also accumulates in the LM of MEs, because ILV formation is compromised. These findings indicate that whether or not Notch is recycled probably depends on the tissue, the stage at which the endosomal pathway is interrupted and which endosomal process is interrupted.

An interesting aspect of our work is that N^K2R^-HA is much more active than N-HA. Our analysis revealed that, while both receptor variants can be activated by the ligands, two additional mechanisms contribute to the enhanced activity of N^K2R^-HA: It is constitutively activated in a ligand-independent manner and the resulting NICD^K2R^-HA is less efficiently degraded than NICD-HA. Thus, besides being required for ILV incorporation, ubi is needed for the efficient degradation of NICD. Although not tested here, it has been shown that the stability of NICD is a critical factor for its activity [56].

How the observed ligand-independent activation of N^K2R^-HA occurs is not clear, but our results suggest that at least part of the activation is connected to the accumulation of N^K2R^-HA in the LM of the ME. This accumulation is the major difference between N-HA and N^K2R^-HA we have detected. It is also observed in other mutants where activation of the N pathway in a ligand-independent manner is observed. Examples are mutants of the ESCRT machinery and also the tumour suppressor gene *lethal (2) giant discs* (*lgd*). In these cases, the function of the ESCRT machinery is disturbed and it has been proposed that a fraction of N remains in the LM, which is subsequently activated [10, 16, 57]. We here show that N^K2R^-HA is not incorporated into ILVs, because it is not being recognised by the ESCRT machinery. Moreover, co-expression of Su(dx), which promotes the incorporation of Notch into ILVs, reduces the activity of N^K2R^-HA. Previous work indicates that this Su(dx)-mediated incorporation into ILVs is mediated by the ESCRT machinery [22, 43]. Together, these findings strongly support the notion that N that remains in the LM, either because of inactivation of the ESCRT function, or removal of the Ks in its ICD, or loss of Su(dx) function, leads to uncontrolled ligand-independent activation. It has been proposed that the activation of the fraction of Notch that remains at the LM occurs via alternative ecto-domain shedding [10]. The acidification of the endosome is an ongoing process during endosomal maturation, and shortly before fusion with the lysosome, the ME is as acidic as the lysosome. The acidified environment in turn activates the acidic hydrolases, which digests the ECD of Notch in the fraction in the LM. Thus, alternative ecto-domain shedding by the luminal hydrolases is initiated in the MEs at the end of their lifetime. The alternative ecto-domain shedding causes the release of NICD via γ-secretase cleavage. The extension of lifetime of the MEs of N^K2R^-HA expressing cells caused by the loss of Rab7 probably enhances alternative ectodomain shedding, because it allows the continuation of acidification. Hence, it is pivotal to precisely regulate the amount of N in the LM of the ME for the regulation of the ligand-independent activity of the N pathway. The finding that N^K2R^-HA is activated in a ligand-independent manner refrained us from trying to generate a N^K2R^-HA variant in the endogenous N locus by gene editing. We suspected that that the connected uncontrolled activation of the N pathway during development would prevent the survival of transformed flies.

One question is how N^K2R^-HA is degraded? Indeed, our experiments show that expression of N^K2R^-HA causes the endosomes to lose Rab7 and become GVs. Since Rab7 is required for the fusion with the lysosome, we do not think that a fusion of GVs with the lysosome occurs to a large extent to degrade N^K2R^-HA in this compartment. We believe that the activation of N^K2R^-HA by alternative ectodomain shedding of N^K2R^ in the GVs also initiates its degradation [10]. It is followed by the release of the NICD^K2R^-HA via γ-secretase cleavage. Our pulse-chase experiments show that also NICD^K2R^-HA is eventually degraded in an ubi-independent manner. Thus, the uncontrolled activation also leads to degradation of N^K2R^-HA.

Another question is why does the accumulation of N^K2R-LL2AA^-HA and N^LL2AA^-HA at the plasma membrane do not affect signalling. One important result of this work is that several endocytic pathways exist. Thus, one explanation is that they might function redundantly. If one pathway is interrupted, the others compensate and no signalling defect can be observed. Another possibility is that two qualitatively different pathways exist, one for bulk endocytosis, which is irrelevant for signalling and a second one that is essential for it. This situation has been observed for the ligands of Notch [58, 59]. In this case, only a small fraction of the ligands enters the pathway relevant for signalling. It is possible that a similar division exists for Notch, which is permanently endocytosed in a ligand-independent manner [10]. The di-leucine motif and ubi might be only required for this bulk endocytosis of Notch. Future work should focus on the characterisation of the various endocytic routes Notch.

## Conclusions

Our analysis reveals that Notch is endocytosed in multiple ways and that ubiquitylation of Notch is not essential for its endocytosis, or its ligand-dependent activation. While ubiquitylation plays a minor role in endocytosis, it is important for the incorporation of endocytosed Notch into the intraluminal vesicles of the maturing endosomes and the degradation of the activated form of Notch in the nucleus. In this way, it prevents uncontrolled ligand-independent activation of Notch and determines the duration of the Notch signal.

## Methods

Key resources table

**Table:** 

Reagent type (species) or resource	Designation	Source or reference	Identifiers	Additional information
Genetic reagent (*D. melanogaster*)	UAS-N-HA (II.) and (III.)	This paper		C-terminally HA-tagged *D. melanogaste*r N receptor. GAL4/UAS-driven expression
Genetic reagent (*D. melanogaster*)	pMT-N-HA	This paper		C-terminally HA-tagged *D. melanogaste*r N receptor. CuSO_4_ inducible pMT promoter.
Genetic reagent (*D. melanogaster*)	UAS-NK2R-HA (II.) and (III.)	This paper		C-terminally HA-tagged *D. melanogaster* N receptor. 27 intracellular lysines replaced by arginines (K2R). GAL4/UAS-driven expression
Genetic reagent (*D. melanogaster*)	pMT-NK2R-HA	This paper		C-terminally HA-tagged *D. melanogaster* N receptor. 27 intracellular lysines replaced by arginines (K2R). CuSO_4_ inducible pMT promoter.
Genetic reagent (*D. melanogaster*)	UAS-NICD-HA (III.)	This paper		C-terminally HA-tagged intracellular domain of the *D. melanogaster* N receptor. GAL4/UAS-driven expression
Genetic reagent (*D. melanogaster*)	UAS-NICDNK2R-HA (III.)	This paper		C-terminally HA-tagged intracellular domain of the *D. melanogaster* N receptor. 27 intracellular lysines replaced by arginines (K2R). GAL4/UAS-driven expression
Genetic reagent (*D. melanogaster*)	UAS-NLL2AA-HA (III.)	This paper		C-terminally HA-tagged *D. melanogaste*r N receptor. Di-leucine motif replaced by alanines (LL2AA). GAL4/UAS-driven expression
Genetic reagent (*D. melanogaster*)	UAS-NK2R-LL2AA-HA (III.)	This paper		C-terminally HA-tagged *D. melanogaster* N receptor. 27 intracellular lysines replaced by arginines (K2R). Di-leucine motif replaced by alanines (LL2AA). GAL4/UAS-driven expression
Genetic reagent (*D. melanogaster*)	UAS-dx (II.)	Gift from M. Baron, University of Manchester, UK		GAL4/UAS-driven expression
Genetic reagent (*D. melanogaster*)	UAS-Su(dx) (II.)	Gift from M. Baron, University of Manchester, UK		GAL4/UAS-driven expression
Genetic reagent (*D. melanogaster*)	hh-GAL4	Bloomington Drosophila Stock Center	BL67046	
Genetic reagent (*D. melanogaster*)	tub-GAL80^ts^	Bloomington Drosophila Stock Center	BL7108	
Genetic reagent (*D. melanogaster*)	tub-GAL4	Bloomington Drosophila Stock Center	BL 5138	
Genetic reagent (*D. melanogaster*)	ptc-GAL4	[60]		
Genetic reagent (*D. melanogaster*)	UAS-GFP	[61]		GAL4/UAS-driven expression
Genetic reagent (*D. melanogaster*)	FRT82B ubi-nls-RFP	Bloomington Drosophila Stock Center	BL30555	
Genetic reagent (*D. melanogaster*)	NRE-GFP (II.)	[24]		
Genetic reagent (*D. melanogaster*)	NRE-nlsGFP (Gbe+Su(H)-nlsGFP) (III.)	[62]		
Genetic reagent (*D. melanogaster*)	N-YFP (X)	[41, 42]		Endogenously tagged N
Genetic reagent (*D. melanogaster*)	Dl^rev10^ Ser^Rx82^ e FRT82B	[63]		
Genetic reagent (*D. melanogaster*)	kuz^ES24^ FRT40A	[28]		
Genetic reagent (*D. melanogaster*)	Psn^C1^ FRT2A	[32]		
Genetic reagent (*D. melanogaster*)	tub-Rab5-CFP tub-Rab7-YFP (III.)	[64]		
Genetic reagent (*D. melanogaster*)	NRE-Fluc	[65]		
Antibody	anti-β-Gal (rabbit polyclonal)	Cappel		IF (1:5000)
Antibody	anti-HA (rat monoclonal)	Roche	3F10	IF (1:500) WB (1:2000)
Antibody	anti-HA (rabbit monoclonal)	Cell Signaling Technology	C29F4	IF (1:2000) WB (1:5000)
Antibody	anti-Rab5 (mouse monoclonal)	Abcam	ab91261	IF (1:500)
Antibody	anti-Calnexin99 (mouse monoclonal)	DSHB	6-2-1	IF (1:100)
Antibody	anti-Golgin84 (mouse monoclonal)	DSHB	12-1	IF (1:100)
Antibody	anti-Wg (mouse monoclonal	DSHB	4D4	IF (1:250)
Antibody	anti-alpha-Catenin (mouse monoclonal)	DSHB	DCAT-1	WB (1:2000)
Antibody	anti-Peanut (mouse monoclonal)	DSHB	4C9H4	WB (1:2000)
Antibody	anti-Flag (mouse monoclonal)	Sigma	M2	WB (1:2000)
Antibody	anti-NECD (mouse monoclonal)	DSHB	C458.2H	IF: (1:100)
Antibody	anti-Dx (rat monoclonal)	[66, 67]		IF: (1:50)
Antibody	anti-Su(dx) (rabbit polyclonal)		SK289	IF (1:500)
Antibody	anti-mouse Alexa 568 (goat polyclonal)	Invitrogen		IF (1:500)
Antibody	anti-mouse Alexa 647 (goat polyclonal)	Invitrogen		IF (1:500)
Antibody	anti-mouse Alexa 488 (goat polyclonal)	Invitrogen		IF (1:500)
Antibody	anti-rat Alexa 568 (goat polyclonal)	Invitrogen		IF (1:500)
Antibody	anti-rat Alexa 647 (goat polyclonal)	Invitrogen		IF (1:500)
Antibody	anti-rat Alexa 488 (goat polyclonal)	Invitrogen		IF (1:500)
Antibody	anti-rabbit Alexa 568 (goat polyclonal)	Invitrogen		IF (1:500)
Antibody	anti-rabbit Alexa 647 (goat polyclonal)	Invitrogen		IF (1:500)
Antibody	anti-rabbit Alexa 488 (goat polyclonal)	Invitrogen		IF (1:500)
Antibody	anti-rat HRP (goat polyclonal)	Jackson Immuno Research		WB (1:5000)
Antibody	anti-mouse HRP (goat polyclonal)	Jackson Immuno Research		WB (1:5000)
Antibody	anti-mouse IRDye 800CW (goat polyclonal)	Abcam	ab216772	WB (1:5000)
Antibody	anti-rabbit IRDye 680RD (goat polyclonal)	Abcam	ab216777	WB (1:5000)
Other	Hoechst 33258	Sigma Aldrich/Merck	B2883	IF (1:10000), DNA/Nucleus staining
Sequence-based reagent	NICD-HA ATG EcoRI for	This paper	PCR primer	AAAGAATTCATGCGCAAGCGCC GCCGCCAGCA
Sequence-based reagent	NICD-HA rev	This paper	PCR primer	CTCTTGGCGGCATCTGCTCG
Sequence-based reagent	NICD^K2R^-HA ATG EcoRI for	This paper	PCR primer	AAAGAATTCATGCGCCGCCGCC GCCGCCAGCA
Sequence-based reagent	NICD^K2R^-HA rev	This paper	PCR primer	CTGCGGGCGGCATCTGCTCG
Sequence-based reagent	QC di-leucine LL2AA for	This paper	PCR primer	TCACGATATTGTCAGGGCCGCC GACGAGCATGTGCCGC
Sequence-based reagent	QC di-leucine LL2AA rev	This paper	PCR primer	GCGGCACATGCTCGTCGGCGGC CCTGACAATATCGTGA

### Drosophila stocks and genetics

A complete list of all stocks used in this study is found in the key resources table above. Flies were raised on standard cornmeal/molasses/yeast diet and kept at room temperature. Crossings were raised on 25 °C, except for experiments containing the temperature-sensitive tub-Gal80^ts^ [23]. Those flies were kept at 18 °C (restrictive temperature) to inhibit Gal4/UAS-mediated expression [68] and shifted to 29 °C (permissive) for specific time spans to activate UAS-based expression. Flp/FRT system [69] induced clones were either generated by expression of UAS-FLP or using hs-FLP with a 70-min heatshock in the first instar larval stage (24–48 h after egg laying).

### Generation of transgenic flies

For generation of UAS expressed N-HA and NK2R-HA constructs, the coding region of N was used to synthesise N-HA and NK2R-HA DNA fragments flanked by EcoRI and XbaI restriction sites. c-DNA was synthesised by GenScript®. N-HA and NK2R-HA c-DNA were restriction digested either with EcoRI and BglII as well as BglII and XbaI in a separated approach. The two DNA fragments of both constructs were subsequently cloned into pUAST-attB vector, which was restriction digested by EcoRI and XbaI.

N-HA and NK2R-HA were additionally subcloned into pMT vector (pMT-N-HA and pMT-NK2R-HA) to express N constructs in S2 cells.

For generation of UAS expressed NICD-HA and NICDK2R-HA constructs, N-HA and NK2R-HA were used as PCR template. PCR primers were designed to exclusively amplify the intracellular domains flanked by EcoRI and XbaI (NICD-HA primer: NICD-HA ATG EcoRI for / NICD-HA rev) (NICDK2R-HA primer: NICDK2R-HA ATG EcoRI for / NICD^K2R^-HA rev). The PCR fragments were restriction digested by EcoRI and XbaI and subsequently cloned into pUAST-attB vector.

For generation of UAS expressed NLL2AA-HA and NK2R-LL2AA-HA constructs, a site directed mutagenesis PCR was performed on pUAST-attB N-HA and pUAST-attB NK2R-HA (Primer: QC di-leucine LL2AA for / QC di-leucine LL2AA rev). The PCR products were restriction digested by BglII and XbaI and subsequently cloned into fresh pUAST-attB N-HA and pUAST-attB NK2R-HA vector. Site-directed mutagenesis PCRs were performed according to the QuikChange II Site-Directed Mutagenesis protocol by Agilent Technologies. All primer sequences are found in the key resources table above.

Generation of transgenic flies was performed by attB/attP-specific genomic integration into the landing sites 51C (for 2nd chromosome) and 86Fb (for 3rd chromosome) [70]. Injection of embryos was either performed in house or by BestGene Inc (CA 91709, USA).

### Immunohistochemistry (IHC)

A complete list of all antibodies used in this study is found in the key resources table above. Late L3 larvae or pupae were dissected in phosphate-buffered saline (PBS, pH 7.4) on ice and immediately fixed for 30 min in 4% paraformaldehyde in PBS. Following three 10-min wash steps in PBT (0.3% Triton X-100 in PBS), tissue was blocked with 5% normal goat serum (NGS, Jackson ImmunoResearch) in PBT and subsequently incubated with primary antibodies in 5% NGS in PBT for 90 min. After three 15-min wash steps with PBT, discs were incubated with fluorochrome-conjugated antibodies (Alexa-488, -568, -647 from Thermo Fisher Scientific) for 60 min in 5% NGS in PBT. For nuclear staining, Hoechst 33258 (Sigma Aldrich) was used at a concentration of 1:10,000. Discs were mounted in Vectashield (Vector Laboratories) and imaged with a Zeiss AxioImager Z1 wide field microscope equipped with a Zeiss Apotome or Zeiss Elyra PS (SR-SIM).

For immunohistochemistry on S2 cells, cells were grown in 12-well plates and reseeded to coated coverslips. Cells were then washed with ice-cold PBS pH 7.4 and fixed for 30 min with 4% paraformaldehyde in PBS at room temperature. After two short wash steps, the cells were permeabilized with 0.2% PBT (0.2% Triton X-100 in PBS) for 15 min at room temperature and subsequently washed for two times with PBS again. The cells were blocked with 0.1% normal goat serum (NGS, Jackson ImmunoResearch) in PBS. Cells were then incubated with primary antibody for 2 h in 0.1% NGS at room temperature. After two short washing steps, the cells were incubated with secondary antibody in 0.1% NGS for 30 min at room temperature. Cells were then washed for two times with PBS and incubated with Hoechst 33258 (Sigma Aldrich). The coverslip was mounted in Vectashield and imaged with a Zeiss AxioImager Z1 wide field microscope. The raw data were deconvoluted with Huygens Professional Software according to automated settings.

### Fluorescence intensity measurements

For fluorescence intensity measurements of NRE-GFP, ROIs of 2843 μm^2^ were assigned with Fiji. Measurements were taken in the ventral anterior and the ventral posterior compartment of third instar larvae wing imaginal discs. The posterior fluorescence intensity was normalised to the anterior fluorescence intensity in each wing imaginal disc.

For fluorescence intensity measurements of basal HA-positive vesicles, ROIs were manually assigned with Fiji, according to the diameter of each vesicle (thickness: 258 μm). The fluorescence intensity profile of each vesicle was calculated with the “plot profile” function of Fiji. In this plot, the highest fluorescence intensity of the vesicle limiting membrane and the lowest fluorescence intensity of the vesicle lumen was identified. The reciprocal difference of these measurements was taken as a value to represent how much receptor is inside the vesicle lumen in relation to the vesicle limiting membrane.

For fluorescence intensity measurements of N constructs at the apical plasma membrane, ROIs of 2267 μm^2^ were assigned with Fiji. The fluorescence intensity of the focal plane with the highest membrane signal was measured and subsequently deleted from the Z-stack. The remaining focal planes of the Z-stack were transformed into a maximum intensity projection (MIP). The ratio of the fluorescence intensity of the apical membrane and the MIP was taken as a value to describe how much receptor is localised at the apical membrane. All statistical analyses were performed with GraphPad Prism 7.0 d.

### S2 cell culture

S2 cells were grown in Schneider’s Medium (Pan Biotech) with 10% FBS (P30-1502, Pan Biotech) and 1% penicillin-streptomycin (Invitrogen) at 25 °C. Transfection was performed by using Effectene (Qiagen) according to the standard protocol. Expression of the constructs was regulated by the CuSO_4_ inducible pMT promoter (Invitrogen).

### N uptake assay

For N-HA and NK2R-HA uptake in S2 cells, UAS-N-HA or UAS-NK2R-HA and pMT-Gal4 were transfected and expressed for 24 h at 25 °C. The cells were then transferred to coated coverslips and subsequently incubated for 15 min with anti-NECD antibody on ice. Cells were washed with ice-cold Schneider’s medium and chased for 45 min or 90 min at 25 °C. At time point 0 min, 45 min and 90 min, the cells were fixed, permeabilized and stained as described earlier.

### Ubiquitination assay

Ubiquitination assay was carried out as previously described [19].

To analyse Su(dx)-induced Notch ubiquitination, S2 cells were seeded and transfected with pMT-Notch-HA, pMT-Flag-Ubiquitin and pMT-Su(dx) in a 6-well plate using Effectene (Qiagen) at 25 °C. After 48 h at 25 °C, gene expression from the pMT promoter was induced with 1 mM CuSO_4_ at 18 °C for 24 h. Then the cells were treated by 50 μM MG132 (Enzo Life Sciences), 200 μM chloroquine (Sigma) and 10 mM NH_4_Cl at 18 °C for 1 h, and then transferred to 25 °C for 15 min.

For Notch ubiquitination by Dx, S2 cells were transfected with pMT-Notch-HA, pMT-Flag-Ubiquitinmono and pMT-Dx-V5 for 48 h, and the pMT expression was induced with 1 mM CuSO_4_ at 18 °C for 24 h.

Cell lysates were extracted in lysis buffer (50 mM Tris-HCl (pH 7.5), 125 mM NaCl, 5% glycerol, 0.2% NP-40, 1.5 mM MgCl_2_, 1 mM DTT, 1 mM EDTA, 50 μM MG132 proteasome inhibitor, Halt Protease and Phosphatase Inhibitor Cocktail (Thermo Scientific), 5 mM N-Ethylmaleimide) and HA-tagged Notch proteins were pulled down with Monoclonal (HA-7) anti-HA Agarose (Merck).

Protein samples were separated on 3–8% Nupage Tris-Acetate gels (Thermo Fisher Scientific) and transferred to PVDF membrane (Merck). Primary antibodies used for western blotting were Rabbit monoclonal (C29F4) anti-HA (Cell Signaling Technology), Mouse monoclonal (M2) anti-Flag (Merck) and Mouse monoclonal (4C9H4) anti-Peanut (Developmental Studies Hybridoma Bank). IRDye 800CW-conjugated anti-Mouse IgG and IRDye 680RD-conjugated anti-Rabbit IgG (Abcam) were used to capture the gel images on the Odyssey CLx imaging system (LI-COR Biosciences).

### Luciferase reporter assay

For luciferase reporter assay, S2 cells were grown in 12-well plates and transfected with pMT-N-HA or pMT-NK2R-HA, NRE-Fluc and act-Rluc. For Dl- or Dx-induced N signalling, the cells were additionally transfected with pMT-Dl or pMT-dx and pMT-Su(dx). Expression was induced for 24 h at 25 °C. The cells were subsequently reseeded in 96-well plates. Luciferase activity was assayed with Dual-Glo Luciferase (Promega) according to the standard protocol and quantified by luminometer (Berthold). Firefly luciferase activity was normalised to Renilla luciferase activity.

## Supplementary information

**Additional file 1: Figure S1.** Luciferase-Assay to analyse the activation of N-HA and N^K2R^-HA by Dl and Dx in S2 cells. N activity in S2 cells was revealed by a luciferase assay using NRE-Luciferase (see M&M). (A) N-HA and N^K2R^-HA activation by Dl. The activation was normalised to basal N activity of the control (*n*=3 luciferase-assays). The N-HA activity was increased by co-expression of Dl, indicating that N-HA can be activated in a ligand- dependent manner. Likewise, N^K2R^-HA was further activated by Dl, indicating that beside the ligand-independent activation, it can be also activated by its ligands.

**Additional file 2: Figure S2.** Emergence of GVs as a result of longer expression of N^K2R^-HA. While only a few GVs are observed upon a pulse of 14, 5 h expression, the number dramaticly increased if the pulse were extended to 24 h.

**Additional file 3: Figure S3.** N-HA and N^K2R^-HA ubiquitination assay. (A, B) Ubiquitination-assay of N-HA and N^K2R^-HA. The variants were co-expressed in S2 cells with Flag tagged Ubiquitin (Flag-Ubi) and Su(dx) (A) or Dx (B) (n= 3). Full length N-HA and N^K2R^-HA bands run at 300 kDa, that of the activated intracellular domains at 110 kDa. The results show that N-HA is ubiquitinated by Su(dx) (upper left panel), as well as Dx (lower left panel). In contrast, N^K2R^-HA was not ubiquitinated by Su(dx) (upper left panel) or Dx (lower left panel). Right panels show whole cell extracts. Peanut antibody staining was used as loading control. All samples used for the IP showed a similar protein level.

**Additional file 4: Figure S4. **Impact of Su(dx) on the endocytosis and activation of endogenous N. Su(dx) was constitutively expressed under control of *hh-GAL4* in the posterior compartment of third instar larvae wing imaginal discs. Expression was performed at 29°C. The localisation of endogenous N was revealed by anti-NECD antibody staining, the activity of the pathway by expression of NRE-GFP. (A) Expression of Su(dx) caused the formation of a small scar at the expression domain boundary (arrow) and a suppression of the expression of the activity reporter NRE-GFP (arrow), indicating that it suppresses the activity of the pathway. (C) Irrespective of Su(dx) over-expression, endogenous N clearly localised at the apical plasma membrane, indicated by the honeycomb pattern (arrows in the upper panels). This result indicates that the constitutive over-expression of Su(dx) had no obvious impact on endocytosis of endogenous N. Upper panels show a Z- projection of the A/B axis. The apical plasma membrane is indicated by arrows. The dashed yellow line highlights the expression boundary. Lower panels show a focal plane of the apical plasma membrane. Due to the scar formation of the wing imaginal discs, the apical plasma membrane of the anterior and posterior compartment is slightly shifted in its focal plane. Therefore, (C) shows the focal plane of the apical plasma membrane of the anterior control compartment, while (C’) shows the apical domain of the posterior Su(dx) expressing compartment. (D) Dx over-expression resulted in a formation of enlarged N positive vesicles (magnification in insert). (B) In contrast, Su(dx) had no obvious impact on endogenous N vesicle formation.

**Additional file 5: Figure S5. **Localisation of N^LL2AA^-HA and N^K2R-LL2AA^-HA on basal vesicles. N^LL2AA^-HA and N^K2R-LL2AA^-HA were expressed under control of *hh-GAL4 tub-GAL80*^*ts*^ for 14.5 h in the posterior compartment of third instar larvae wing imaginal discs. (A) N^LL2AA^-HA localised in the lumen of the basally located vesicles. (B) In contrast, N^K2R-LL2AA^-HA was mainly localised in the limiting membrane.

## Data Availability

All data generated or analysed during this study are included in this published article and its supplementary information files. Additionally, raw images and data files are available within the following figshare repositories: Luciferase assay (10.6084/m9.figshare.17064374), Notch Blot (10.6084/m9.figshare.17054120), Surface intensity measurements (10.6084/m9.figshare.17054114), GV intensity measurements (10.6084/m9.figshare.17054078).
